# *TP73* Isoform-specific disruption reveals a critical role of TAp73beta in growth suppression and inflammatory response

**DOI:** 10.1038/s41419-022-05529-7

**Published:** 2023-01-11

**Authors:** Jin Zhang, Wenqiang Sun, Wensheng Yan, Xiangmudong Kong, Tong Shen, Kyra Laubach, Mingyi Chen, Xinbin Chen

**Affiliations:** 1 Comparative Oncology Laboratory, Schools of Veterinary Medicine and Medicine, UC Davis, California Davis, USA; 2 West Coast Metabolomics Center, UC Davis, Califronia Davis, USA; 3 Department of Pathology, Southwestern Medical Center, University of Texas, Dallas, USA; 4 Present Address: Department of Animal Science and Technology, Sichuan Agricultural University, Ya’an, China; 5 Present Address: Berkeley Regional Lab, Pathology/Lab-Histology Department, The Permanente Medical group, Berkeley, CA 94085 USA

**Keywords:** Cell death, Cancer

## Abstract

*TP73* is expressed as multiple N- and C-terminal isoforms through two separate promoters or alternative splicing. While N-terminal p73 isoforms have been well studied, very little is known about p73 C-terminal isoforms. Thus, CRISPR was used to delete *TP73* Exon13 (E13-KO) to induce p73α to p73β isoform switch. We showed that E13-KO led to decreased cell proliferation and migration and sensitized cells to ferroptosis, which can be reverted by knockdown of TAp73β in E13-KO cells. To understand the biological function of p73β in vivo, we generated a mouse model in that the *Trp73* E13 was deleted by CRISPR. We showed that p73α to p73β isoform switch led to increased cellular senescence in mouse embryonic fibroblasts. We also showed that *E13*-deficient mice exhibited shorter life span and were prone to spontaneous tumors, chronic inflammation and liver steatosis as compared to WT mice. Additionally, we found that the incidence of chronic inflammation and liver steatosis was higher in *E13*-deficient mice than that in *Trp73*-deficient mice, suggesting that p73β is a strong inducer of inflammatory response. Mechanistically, we showed that TAp73β was able to induce cysteine dioxygenase 1 (CDO-1), leading to cysteine depletion and subsequently, enhanced ferroptosis and growth suppression. Conversely, knockdown of CDO-1 was able to alleviate the growth suppression and ferroptosis in E13-KO cells. Together, our data suggest that at a physiologically relevant level, TAp73β is a strong inducer of growth suppression but insufficient to compensate for loss of TAp73α in tumor suppression due to aberrant induction of inflammatory response and liver steatosis.

## Introduction

p73, along with p53 and p63, constitute the p53 family of tumor suppressors [1, 2]. Like other family members, *TP73* is expressed as multiple isoforms through alternative promoter usage and splicing. The usage of two separate promoters generates TAp73 and ΔNp73 variants that only differ in their N-terminal transactivation (TA) domain. As the TA domain is conserved in p53, thus, TAp73 is thought to function as a tumor suppressor by regulating an array of genes for growth suppression. By contrast, ΔNp73 does not contain the TA domain and is thought to have oncogenic potential through antagonizing TAp73/p63 and p53. At the C-terminus, TP73 produces at least six alternatively spliced isoforms (α, β, γ, ε, δ, ζ). Among them, TAp73α is the longest form of the p73 protein, which contains a sterile alpha motif (SAM) domain and a transcription-inhibition domain (TID), whereas TAp73β is a smaller polypeptide and considered to be the most active isoform due to lack of the TID and most of the SAM. When over-expressed, TAp73β can elicit growth suppression by inducing many target genes similarly as p53 [3–5].

The biological functions of p73 have been studied in vitro and in vivo, with focus mainly on its N-terminal isoforms. For example, *Trp73*-KO mice that lack all p73 isoforms are runty and have severe neurological and immunological defects, such as hydrocephalus, hippocampal dysgenesis, abnormalities of the pheromone sensory pathway, and chronic infections and inflammation [6]. Similar to *Trp73*-KO mice, ΔNp73-KO mice are prone to delayed onset of moderate neurodegeneration [7]. Additionally, ΔNp73-KO mice are viable and fertile and do not develop spontaneous tumors but show enhanced DNA damage response [8], suggesting a role of ΔNp73 in the DNA damage response pathway. By contrast, TAp73-KO mice are infertile and prone to spontaneous and chemical carcinogen-induced tumors [9–11]. TAp73-KO mice also exhibit a failure of airway multiciliogenesis, lack of mucus clearance, and severe respiratory tract infections [12–14]. These in vivo studies clearly demonstrate that TAp73 is a bona fide tumor suppressor while ΔNp73 has an oncogenic potential.

Unlike the N-terminal isoforms, the biological functions of p73 C-terminal isoforms remains largely uncharacterized. Intriguingly, early studies have shown that p73 C-terminal isoforms, including p73β, are frequently up-regulated in human cancers such as breast and colon cancers [15, 16]. However, whether and how these isoforms contribute to tumorigenesis remains unknown. It should be mentioned that due to their relative low abundance, most studies were carried out in cell culture with an ectopic expression system to investigate p73 C-terminal isoforms. Although these studies suggest that p73 C-terminal isoforms have distinct activities in terms of transcriptional regulation and target gene selection, a system with more physiological relevance is needed to study the biological function of p73 C-terminal isoforms and their correlations with human diseases. In this regard, CRISPR was used to introduce *TP73* exon 13 (E13) skipping in human cancer cell lines and in mice. We showed that *E13* skipping leads to isoform switch from p73α to p73β and subsequently, enhances p73β protein expression. Importantly, we found that when expressed at a physiologically relevant level, TAp73β is a potent inducer of growth suppression and inflammatory response in vitro and in vivo. We also found that cysteine dioxygenase 1 (CDO-1) is a novel target of TAp73β and mediates TAp73β-induced growth suppression and inflammatory response by regulating the level of intracellular cysteine.

## Materials and Methods

### Reagents

Scrambled siRNA (5’- GGC CGA UUG UCA AAU AAU U -3’), p73α/β siRNA (5’-UCC UCU CGC CCA UGA ACA A-3’), and CDO1 siRNA (5’-GCG AUG AGG UCA AUG UAG A-3’) were purchased from Dharmacon (Chicago, IL). For siRNA transfection, RNAiMax (Life Technologies) was used according to the user’s manual. Proteinase inhibitor cocktail and Erastin was purchased from Sigma-Aldrich (St. Louis, MO). Magnetic Protein A/G beads were purchased from MedChem (Santa Clara, CA). RiboLock RNase Inhibitor and Revert Aid First Strand cDNA Synthesis Kit were purchased from Thermo Fisher (Waltham, MA).

### Plasmids

pcDNA3-HA-TAp73α and pcDNA3-HA-TAp73β were described previously [17]. To generate a vector expressing a single guide RNA (sgRNA) that targets TAp73, ΔNp73 and E13, two oligos were annealed and then cloned into the pSpCas9(BB) sgRNA expression vector [18] via BbsI site. To generate TAp73 sgRNA vector, the oligo sequences were 5’-ACC GCT TCC CCA CGC CGG CCT CCG-3’ and 5’-AAA CCG GAG GCC GGC GTG GGG AAG C-3’. To generate ΔNp73 sgRNA vector, the oligo sequences were 5’-CAC CGT ACA GCA TGG TAG GCG CCG-3’ and 5’-AAA CCG GCG CCT ACC ATG CTG TAC-3’. To generate vector expressing E13 sgRNA#1, the oligo sequences were 5’- CAC CG CAG CAT TAG CTT CCG AGC AC-3’, and 5’-AAA CGT GCT CGG AAG CTA ATG CTG C-3’. To generate vector expressing E13 sgRNA#2, the oligo sequences were 5’- CAC CGT GAA ATA CTC GAT GCA GTT-3’ and 5’-AAA CAA CTG CAT CGA GTA TTT CAC-3'.

### Mice and mouse embryonic fibroblasts (MEFs) isolation

*Trp73*^*+/−*^ mice were generated as described previously [19]. *E13*^*+/−*^ mice were generated by the Mouse Biology Program at University of California at Davis. All animals and use protocols were approved by the University of California at Davis Institutional Animal Care and Use Committee. To generate WT and *E13*^*−/−*^ MEFs, *E13*^*+/−*^mice were bred and MEFs were isolated from 12.5 to 13.5 postcoitum (p.c.) mouse embryos as described previously [20]. MEFs were cultured in DMEM supplemented with 10% FBS (HyClone), 55 μM β-mercaptoethanol, and 1× non-essential amino acids (NEAA) solution (Cellgro). The genotyping primers for the WT allele were forward primer 5’-GAG TGC TTC ACT TCC CAA GGG TTG C-3’ and reverse primer 5’-CCT GGA AAC AAC CCT GCC TAA GAA ATC-3’. The genotyping primers for the *E13*-KO allele were forward primer 5’-GCA CTA GAT GGT TAG GCA CTG AGG-3’ and reverse primer 5’-GGA GGC TCT AAC AAG AGA GAA AGC TC-3’.

### Cell culture, cell line generation

H1299 and Mia-PaCa2 cells were obtained from NCI Developmental Therapeutics Program (DTP). Because all cell lines from NCI DTP have been thoroughly tested and authenticated, we did not authenticate the cell lines used in this study. Cells were tested negative for mycoplasma after thawing and used within 2 months. H1299 and Mia-PaCa2 cells and their derivatives were cultured in DMEM (Dulbecco’s modified Eagle’s medium, Invitrogen) supplemented with 10% fetal bovine serum (Hyclone). To generate E13-KO cell lines, H1299 or Mia-PaCa2 cells were transfected with pSpCas9(BB)-2A-Puro vector expressing a sgRNA, and then selected with puromycin for 2–3 weeks. Individual clone was picked confirmed by sequence analysis or western blot analysis. The genotyping primers for sequencing p73 exon 13 were forward primer 5’-TGG CCA CCT GTG GGC TGG-3’ and a reverse primer 5’-GTT ACT CAA TGG TCA GGT TC-3’.

### Western blot analysis

Western blot analysis was performed as previously described [21]. Briefly, whole cell lysates were harvested by 2×SDS sample buffer. Proteins were separated in 7–13% SDS-polyacrylamide gel, transferred to a nitrocellulose membrane, probed with indicated antibodies, followed by detection with WesternBright ECL HRP substrate (Advansta, San Jose, CA) using UVP Chemistudio from Analytik Jena (Upland, CA) and visualized by VisionWorks®LSsoftware. The antibodies used in this study were: anti-Actin (sc-47778, 1:3000), anti-p21 (sc-53870, 1:3000) and anti-PML (sc-377390, 1:3000) from Santa Cruz Biotechnology (Dallas, TX); Anti-TAp73 (A300-126A, 1:2000) from Bethyl Laboratories (Montgomery, TX); Anti-CDO1 from Abcam (Waltham, MA).

### RNA isolation and RT-PCR

Total RNA was isolated with Trizol reagent as described according to user’s manual. cDNA was synthesized with Reverse Transcriptase and used for RT-PCR. The PCR program used for amplification was (i) 94 °C for 5 min, (ii) 94 °C for 45 s, (iii) 58 °C for 45 s, (iv) 72 °C for 30 s, and (v) 72 °C for 10 min. From steps 2 to 4, the cycle was repeated 22 times for actin and GAPDH, 28–35 times depending on the targets. The primers for the actin were forward primer 5’- TCC ATC ATG AAG TGT GAC GT-3’ and reverse primer 5’-TGA TCC ACA TCT GCT GGA AG-3’, the primers to amplify human p73α/β/γ/ε were a forward primer 5’- CAG CAG CAG CAG CTC CTA CA-3’ and a reverse primer 5’- TAC TGC TCG GGG ATC TTC AG -3’, the primers to amplify mouse p73α/β were a forward primer 5’- GCG AGG CCG GGA GAA CTT TGA G-3’ and a reverse primer 5’- TGG CTC TGC TTC AGG TCC TGT AGG C -3’. The primers for the human CDO1 were a forward primer 5’- CTT CTG TGA CCC ACG GCT TCT AAT AGA G-3’ and a reverse primer 5’- GCT GGG CCA TTT AGT CAG TGC ATG-3’. The primers for the human PTGS2 were a forward primer 5’- CTG ATG ATT GCC CGA CTC CC-3’ and a reverse primer 5’- TCG TAG TCG AGG TCA TAG TTC-3’. The primers for the human TFRC were a forward primer 5’- GAG GAG CCA GGA GAG GAC TT-3’ and a reverse primer 5’- ACG CCA GAC TTT GCT GAG TT-3’. The primers for the human LPCAT4 were a forward primer 5’- GCC GGT CTT AGT GAG GAG CAG CTT C-3’ and a reverse primer 5’- ACG GAA AGG TTC TCA GCT CGG GAC-3’.

### ChIP assay

ChIP assay was performed as previously described [22]. Briefly, chromatin was cross-linked in 1% formaldehyde in phosphate-buffered saline (PBS). Chromatin lysates were sonicated to yield 200- to 1,000-bp DNA fragments, immunoprecipitated with a control IgG or an antibody against HA and the DNA-protein immunocomplex were brought down by magnetic protein A/G beads⊡ After reverse cross-linking and phenol-chloroform extraction, DNA fragments were purified, followed by PCR to visualize the enriched DNA fragments. The primers for the CDO1 were a forward primer 5’- CTT CTG TGA CCC ACG GCT TCT AAT AGA G-3’ and a reverse primer 5’- GCT GGG CCA TTT AGT CAG TGC ATG-3’. The primers for the GAPDH were a forward primer 5’- AAA AGC GGG GAG AAA GTA GG-3’ and a reverse primer 5’- AAG AAG ATG CGG CTG ACT GT-3’.

### Colony formation assay

H1299 cells and their derivatives (∼1000 per well) were seeded in triplicate in a six-well plate and then maintained in fresh medium for 2 weeks. The clones were fixed using methanol/glacial acetic acid (7:1) followed by crystal violet staining. To quantify the colony results, Image J software was used with the ColonyArea plugin installed as previously described [23].

### Wound healing assay

2 × 10^5^ cells were seeded in a 6-well plate cells and grown for 24 h. The monolayers were wounded by scraping with a P200 micropipette tip and washed two times with PBS. At indicated time points after scraping, cell monolayers were photographed with phase contrast microscopy. Cell migration was determined by visual assessment of cells migrating into the wounding area. Wound closure percentage was quantified using Image J plugin MRI Wound Healing Tool by comparing the width of the wound between 0 h and indicated time points.

### Cell viability assay

Cell viability was measured by CellTiter-Glo 3D according to manufacturer’s guidelines (Promega). Briefly, 1 × 10^4^ cells were seeded in a 96-well plate cells and grown for 24 h, then the cells were treated DMSO or erastin. At the indicated time points after treatment, cells were added with Titer-Glo reagents and luminescent was measured.

### SA-β-gal staining

The senescence assay was performed as described previously [24]. Briefly, primary MEFs at passage 5 were seeded into a six-well plate and maintained in fresh medium for 72 h. Cells were then washed with PBS and fixed with a fixing solution (2% formaldehyde, 0.2% glutaraldehyde in PBS) for 15 min at room temperature, followed with fresh β-gal staining solution (1 mg/mL 5-bromo-4-chloro-3-indolyl-b-d-galactopyranoside, 40 mM citric acid/sodium phosphate at pH 6.0, 5 mM potassium ferrocyanide, 5 mM potassium ferricyanide, 150 mM NaCl, 2 mM MgCl_2_) overnight at 37 °C without CO_2_. The percentage of senescent cells was calculated as SA-β-gal positive cell divided by the total number of cells counted. Approximately 500 cells were counted.

### Histological analysis

Mouse tissues were fixed in neutral buffered formalin for 18 h and embedded in paraffin blocks. Tissue blocks were sectioned (6 μm) and stained with hematoxylin and eosin.

### Statistical analysis

The Log-rank test was used for Kaplan–Meier survival analysis. Fisher’s exact test and student *t*-test were performed for the statistical analysis. Values of *p* < 0.05 were considered significant. No blinding or randomization was used in animal studies. The variances were similar between the compared groups

## Results

### Loss of E13 leads to increased expression of TAp73β and subsequently, induces growth suppression and sensitizes cells to ferroptosis

**Fig. 1:**
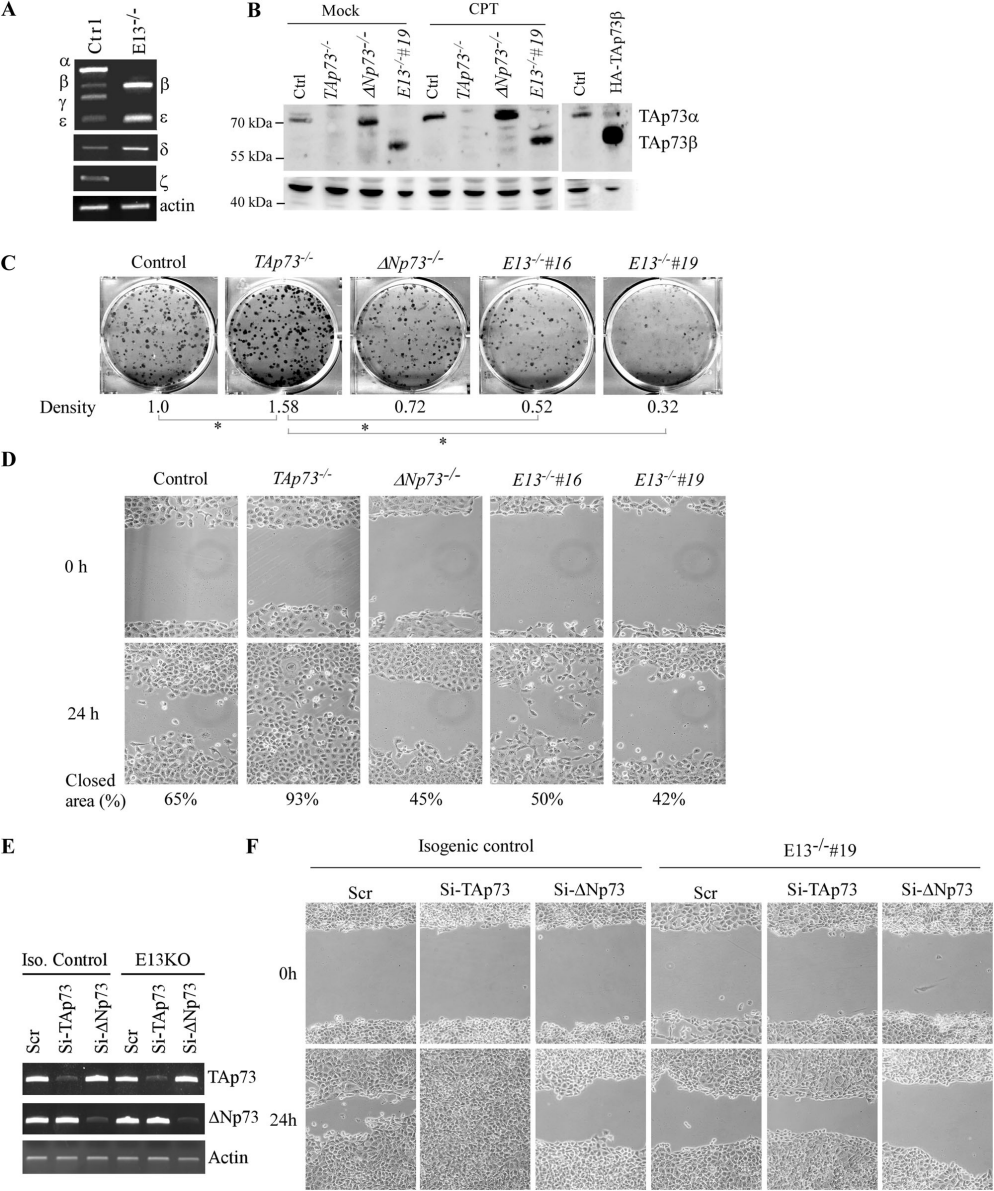
Loss of E13 leads to increased expression of p73β and subsequently, induces growth suppression and sensitizes cells to ferroptosis. **A** The level of various p73 isoforms and actin transcripts was measured in isogenic control and E13-KO H1299 cells. **B** The level of p73 and actin proteins was measured in isogenic control, TAp73-KO, ΔNp73-KO and E13-KO H1299 cells mock-treated or treated with camptothecin (CPT) for 18 h. The HA-tagged TAp73β□□□□□□n were used as a reference control for endogenous TAp73β. **C** Colony formation assay was performed with isogenic control, TAp73-KO, ΔNp73-KO and E13-KO H1299 cells. The relative density was quantified and shown below each image. *p < 0.05 by Student’s *t*-test. **D** Scratch assay was performed with isogenic control, TAp73-KO, ΔNp73-KO and E13-KO H1299 cells. The relative % of wound closure was shown below each image. **E** Isogenic control and E13-KO H1299 cells were transiently transfected with a scrambled siRNA or a siRNA against TAp73 or ΔNp73, followed by RT-PCR to measure the level of TAp73, ΔNp73 and actin transcripts. **F** Scratch assay was performed with cells treated in (E).

To study the biological functions of p73 C-terminal isoforms, we developed a strategy to delete the splicing acceptor for *TP73* exon 13 (E13) using CRISPR-Cas9 (Supplementary Fig. 1A). It is expected that deletion of splicing acceptor for *TP73* E13 would lead to E13 skipping and thereby result in isoform switch from α, γ, and ξ to β, δ, and ε, respectively (Supplementary Fig. 1B). Indeed, we were able to generate stable H1299 and Mia-PaCa2 clones in that *TP73* E13 was excluded, called E13-KO H1299 and Mia-PaCa2 cell lines. Sequence analysis indicated that a total 71 bp deletion, including 43 bp in intron 12 and 28 bp in exon 12, in *TP73* gene in these cells (Supplementary Fig. 1C). Consequently, RT-PCR analyses showed that owing to *TP73* E13 exclusion, p73β became the major isoform expressed in E13-KO H1299 and Mia-PaCa2 cells (Fig. 1A, B and Supplementary Fig. 1C, D). In addition, the level of other isoforms, such as p73ε and p73δ, were increased, however, their relative levels were still much lower when compared with p73β (Fig. 1A, B and Supplementary Fig. 1C, D). Consistent with this, we showed that in TAp73β protein can be detected by an antibody against TAp73 in both E13-KO H1299 and Mia-PaCa2 cells (Supplementary Fig. 1E, F). We would like to note that through the usage of two separate promoters, p73 is expressed as TAp73 and ΔNp73 isoforms, which is known to have opposed functions [25–27]. Thus, to characterize the biological function of p73β, it is important to consider the role of TAp73β and ΔNp73β. To this end, CRISPR-Cas9 method was used to generate two more cell lines, TAp73- and ΔNp73-KO H1299 and Mia-PaCa2 cells in that either TAp73 or ΔNp73 isoforms were deleted. RT-PCR analysis indicated both TAp73 and ΔNp73 transcripts were absent in their respective knockout H1299 cells but was detectable in isogenic control and E13-KO cells (Supplementary Fig. S1G). We would like to note that TAp73 but not ΔNp73 is the major isoform expressed in both H1299 and Mia-PaCa2 cells as revealed by the PCR cycles needed to amplify TA/ΔNp73 transcripts. To verify this, the expression of TAp73 was examined in isogenic control, TAp73-KO, ΔNp73-KO and E13-KO H1299 cells mock-treated or treated with camptothecin, a DNA damaging reagent known to induce TAp73 [28]. We found that TAp73α was absent in TAp73-KO and E13-KO H1299 and Mia-PaCa2 cells but remained detectable in isogenic control and ΔNp73-KO cells (Supplementary Fig. 1H, I), which was further increased by camptothecin treatment (Fig. 1B). Similarly, we found that TAp73b, the major isoform in E13-KO H1299 and Mia-PaCa2 cells, was increased by camptothecin treatment (Fig. 1B and Supplementary Fig. 1F). Together, these data suggested that E13 exclusion led to predominant isoform switch from TAp73α to TAp73β in both H1299 and Mia-PaCa2 cells.

To determine the biological importance of E13-deficiency, colony formation assay was performed with isogenic control, TAp73-KO, ΔNp73-KO, and E13-KO H1299 cells. We found that when compared to isogenic control cells, loss of TAp73 led to increased, whereas loss of ΔNp73 led to moderately decreased, number of colonies in H1299 cells (Fig. 1D), which is consistent with previous reports [29, 30]. Interestingly, the number of colonies was markedly decreased by loss of E13, to an extent greater than that by ΔNp73-KO (Fig. 1C), suggesting that TAp73β inhibits cell proliferation. Next, scratch assay was performed to determine whether E13-KO affects cell migration. We found that loss of TAp73 led to enhanced cell migration whereas loss of ΔNp73 resulted in slower cell migration (Fig. 1D). Importantly, cell migration was suppressed by E13-KO in two independent E13-KO H1299 cell clones (Fig. 1E). To verify that TAp73β inhibits cell migration, TAp73 or ΔNp73 siRNAs were used to knock down TAp73 or ΔNp73 in isogenic control and E13-KO H1299, respectively, followed by cell scratch assay. We found that in isogenic control H1299 cells, knockdown of TAp73 promoted, whereas knockdown of ΔNp73 inhibited, cell migration (Fig. 1E, F), similar to the data obtained in TAp73-KO and ΔNp73-KO H1299 cells (Fig. 1D). Interestingly, we also found that in E13-KO H1299 cell, knockdown of TAp73, but not ΔNp73, attenuated the inhibited cell migration (Fig. 1E, F), suggesting that TAp73β, the major isoform expressed in E13-KO cells, is responsible for the inhibited cell growth and migration.

### Loss of E13 promotes cellular senescence in mouse embryonic fibroblasts

**Fig. 2:**
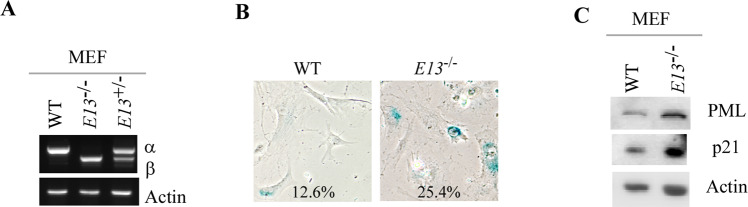
Loss of E13 promotes cellular senescence in mouse embryonic fibroblasts. **A** The level of p73α, p73β, and actin transcripts was measured in WT, *E13*^*+/−*^, and *E13*^*−/−*^ MEFs. **B** WT and *E13*^*−/−*^ MEFs were used for SA-β-gal staining. The percentage of SA-β-gal-positive cells was shown in each panel. **C** The level of PML, p21 and actin proteins was measured in WT and *E13*^*−/−*^ MEFs.

To gain more insight into the growth suppression mediated by E13-deficiency, we generated a mouse model in that Exon 13 in *Trp73* gene was deleted by CRISPR-Cas9 (Supplementary Fig. 2A). The founder mouse showed a deletion of 322 bp (121 bp in intron 12, 94 bp in exon 13, and 107 bp in intron 13) in *Trp73* (Supplementary Fig. 2B). To verify that deletion of E13 in murine *Trp73* gene would elicit the switch from p73α to p73β as that in human cells (Fig. 1), a cohort of WT, *E13*^*+/−*^ and *E13*^*−/−*^ MEFs were generated. Indeed, we found that loss of E13 led to C-terminal isoform switch from p73α to p73β (Fig. 2A), consistent with the observation in human cancer cell lines (Fig. 1). Next, SA-β-Gal staining was performed with WT and *E13*^*−/−*^ MEFs to measure the role of E13 deficiency in cellular senescence, which is considered as a potent tumor suppression mechanism [31]. We found that loss of E13 markedly increased the number of SA-β-gal positive cells (Fig. 2B). We also found that the level of PML and p21, both of which are senescence markers [32, 33], were increased in *E13*^*−/−*^ MEFs as compare to that in WT MEFs (Fig. 2C). Together, these data suggest that E13-deficency promotes tumor suppression by enhancing cellular senescence, likely due to increased p73β expression.

### Mice deficient in E13 have a shortened lifespan and are prone to spontaneous tumors, chronic inflammation and liver steatosis

**Fig. 3:**
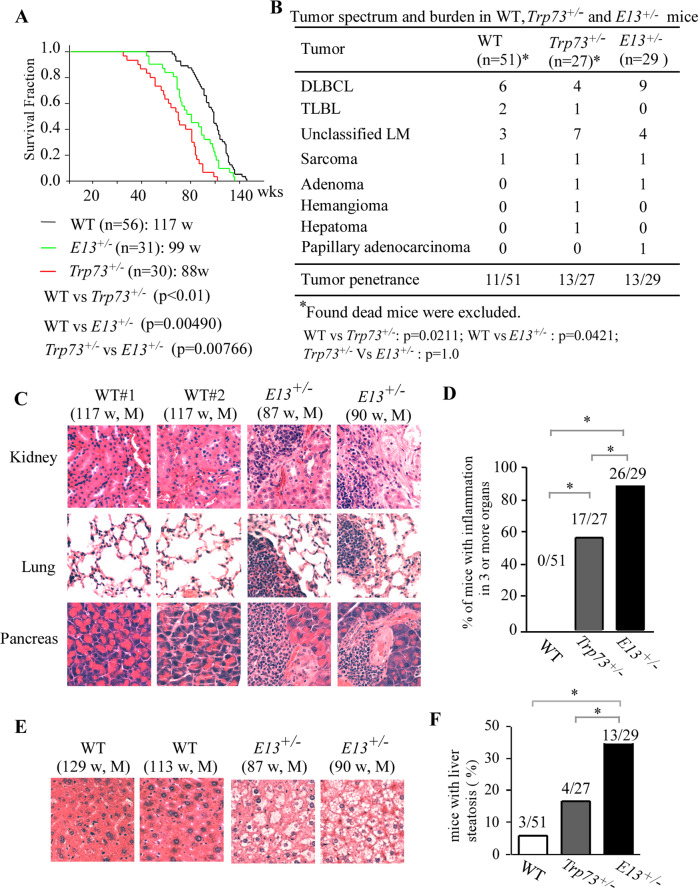
Mice deficient in E13 have a shortened lifespan and are prone to spontaneous tumors, chronic inflammation and liver steatosis. **A** Kaplan-Meyer survival curves of WT (*n* = 56), *Trp73*^*+/−*^ (n = 30), and *E13*^*+/−*^ (*n* = 31) mice. **B** Tumor spectra in WT (n = 56), *Trp73*^*+/−*^ (*n* = 27), and *E13*^*+/−*^ (*n* = 29) mice. **C** Representative images of H.E. stained kidney, lung and pancreas tissues from WT and *E13*^*+/−*^ mice. **D** Percentage of WT, *Trp73*^*+/−*^, and *E13*^*+/−*^ mice with inflammation in 3 or more organs. **E** Representative images of H.E. stained liver tissues from WT and *E13*^*+/−*^ mice. **F** Percentage of WT, *Trp73*^*+/−*^, and *E13*^*+/−*^ mice with liver steatosis.

To determine the biological function of E13 deletion, we generated *E13*^*+/−*^ and *E13*^*−/−*^ mice. We found that similar to *Trp73*^*−/−*^ mice, *E13*^*−/−*^ mice were runty, consistent with the observation from a recent study [34], and thus, not suitable for long term study. In contrast, *E13*^*+/−*^ mice appeared to be normal and thus, a cohort of *E13*^*+/−*^ mice was generated and monitored for potential abnormalities along with WT and *Trp73*^*+/−*^ mice. Additionally, we examined the expression pattern of TAp73 and ΔNp73 in the spleen tissues from E13^+/*−*^ or WT mice, and found that the level of TAp73 and ΔNp73 transcripts were expressed at similar levels in (Supplementary Fig. 3A). To minimize the number of animals used, both WT and 26 out of 30 *Trp73*^*+/−*^ mice, which were generated previously for other studies [19, 35, 36], were used as controls. We would like to mention that all the mice had same genetic background and maintained in the same facility. The median lifespan was 117 weeks for WT mice, 99 weeks for *E13*^*+/−*^ mice and 88 weeks for *Trp73*^*+/−*^ mice (Fig. 3A, Supplementary Tables S1–S3). Statistical analysis indicated that the lifespan for *E13*^*+/−*^ and *Trp73*^*+/−*^ mice was significantly shorter than that for WT mice (Fig. 3A). Notably, the lifespan for *E13*^*+/−*^ mice was longer than that for *Trp73*^*+/−*^ mice (Fig. 3A). Next, histological analyses were performed to examine potential pathological abnormalities among these mice. We found that 11 out of 51 WT mice, 13 out of 27 *Trp73*^*+/−*^ mice, and 13 out of 29 *E13*^*+/−*^ mice developed spontaneous tumors (Fig. 3B). We also examined whether both wt and and E13-KO allele were retained in the tumor samples from *E13*^*+/−*^ mice. We found that these tumors retain both wt and E13-KO allele (Supplementary Fig. 3B). The tumor incidence was significantly higher in both *Trp73*^*+/−*^ and *E13*^*+/−*^ mice than that in WT mice (WT vs *Trp73*^*+/−*^: *p* = 0.0211; WT vs *E11*^*+/−*^: *p* = 0.0421 by Fisher’s exact test). However, there was no difference in the tumor incidence between *Trp73*^*+/−*^ and *E13*^*+/−*^ mice (*p* = 1.0 by Fisher’s exact test). We also noticed that the tumor spectra were very similar between *Trp73*^*+/−*^ and *E13*^*+/−*^ mice, with lymphomas as the most frequent tumors (Fig. 3B). In addition to spontaneous tumors, we found that both *Trp73*^*+/−*^ and *E13*^*+/−*^ mice developed chronic inflammation in multiple organs (Fig. 3C). The percentage of *Trp73*^*+/−*^ and *E13*^*+/−*^ mice with inflammation in 3 or more organs was significantly higher than that in WT mice (Fig. 3D). Interestingly, the incidence of chronic inflammation was even higher in *E13*^*+/−*^ mice than that in *Trp73*^*+/−*^ mice (Fig. 3D). Moreover, we found that *E13*^*+/−*^ mice were prone to liver steatosis (Fig. 3E). Statistical analyses indicated that liver steatosis was significantly higher in *E13*^*+/−*^ mice than that in WT and *Trp73*^*+/−*^ mice (Fig. 3F).

### Loss of E13 promotes ferroptosis

**Fig. 4:**
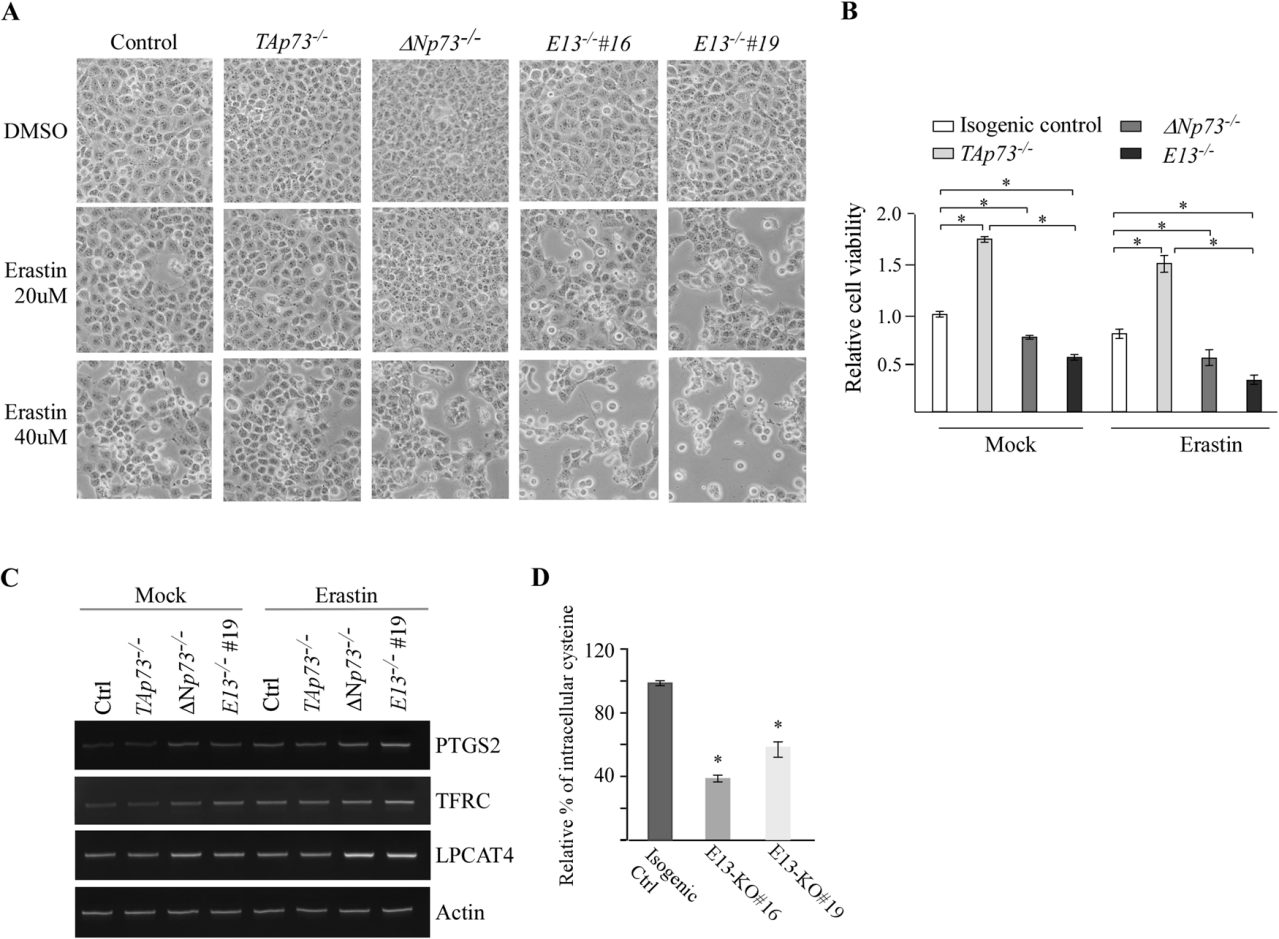
Loss of E13 promotes ferroptosis. **A** Isogenic control, TAp73-KO, ΔNp73-KO and E13-KO H1299 cells were mock-treated or treated with Erastin for 8 h and representative microscopic pictures were taken to show cell morphology. **B** Cells were treated the same as indicated in (**A**), followed by CellTiter-Glo viability assay. Each treatment was performed in triplicates and data were presented as mean ± SEM. *p < 0.05 by student *t*-test. **C** Cells were treated the same as indicated in (**A**), followed by RT-PCR analysis to examine the levels of PTGS2, TFRC, LPCAT4 and actin transcripts. **D** The level of intracellular cystine was measured in isogenic control and E13-KO H1299 cells by Mass Spectrometric analysis. The analysis was performed in triplicates and data were presented as mean ± SEM. *p < 0.05 by student *t*-test.

As shown above, E13-deficient mice were prone to chronic inflammation and liver steatosis when compared to WT or *Trp13*-deficient mice (Fig. 3), suggesting a unique role of p73ß in these processes. In addition, several recent studies suggest that ferroptosis, a type of programmed cell death mediated by iron overload and lipid peroxidation, plays a critical role in the pathological process of non-alcoholic fatty liver disease as well as chronic inflammation [37–39]. Interestingly, p53 was found to exert its tumor suppressive activity by regulating ferroptosis, [40, 41], however, the role of p73 in ferroptosis remains unknown. Thus, we sought to determine whether TAp73, particularly TAp73β, plays a role in ferroptosis since TAp73 protein shares structural and functional similarities with p53. To this end, Erastin, a ferroptosis inducer that inhibit cysteine uptake via system xc − [42], was used to treat isogenic control and TAp73-/ΔNp73-/E13-KO H1299 cells. We would like to note, that H1299 cells are p53-null and thus, would rule out the potential interference of p53 on p73-mediated ferroptosis. It is known that upon treatment with Erastin, ferroptotic cells exhibit distinct morphology changes, such as rounding up and detaching from culture plates [42]. Indeed, we found that while the morphology of isogenic control, TAp73-KO, ΔNp73-KO and E13-KO H1299 cells remained unaltered in the absence of Erastin, isogenic control, ΔNp73-KO and E13-KO H1299 cells, but not TAp73-KO cells, were rounded and detached from the plates in response to Erastin treatment (Fig. 4A). To verify this, CellTiter-Glo viability assays were performed. We found that when compared to isogenic control cells, loss of TAp73 enhanced cell viability whereas knockout of ΔNp73 or E13 inhibited cell growth under mock treatment, consistent with data from colony formation and migration assays (Fig. 1C, D). Importantly, we found that Erastin-induced ferroptosis was suppressed by TAp73-KO but increased by ΔNp73-KO (Fig. 4B), suggesting that an TAp73 promotes whereas ΔNp73 inhibits ferroptosis. Additionally, E13-KO sensitized H1299 cells to Erastin-induced ferroptosis and the sensitivity to Erastin was even greater in E13-KO cells than that in ΔNp73-KO cells (Fig. 4B). To verify this, we examined the expression of three well-defined ferroptosis markers, PTGS2, TFRC, and LPCAT4 [43, 44]. We found that the levels of PTGS2, TFRC, and LPCAT4 transcripts were increased by loss of ΔNp73 or E13-KO in H1299 cells mock-treated or treated with Erastin (Fig. 4C). Furthermore, as Erastin-induced ferroptosis is known to be trigged by cysteine depletion [45], mass spectrometry was performed and showed that loss of E13 markedly decreased the level of intracellular cysteine in H1299 cells (Fig. 4D), consistent with the observation that E13-KO cells showed increased sensitivity to Erastin-induced ferroptosis (Fig. 4A–C). Together, these data indicate that loss of E13 facilitates Erastin-induced ferroptosis.

### TAp73β is required for growth suppression and ferroptosis in E13-KO cells

**Fig. 5:**
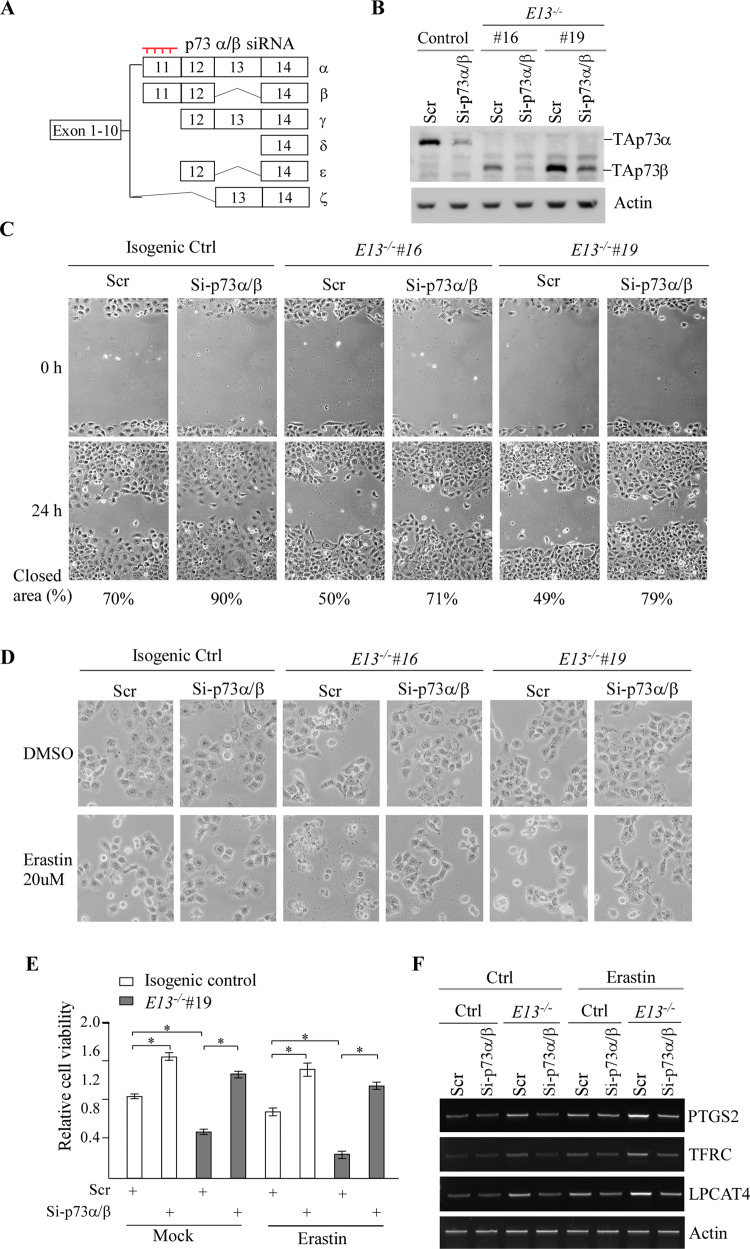
TAp73β is required for growth suppression and ferroptosis in E13-KO cells. **A** Schematic diagram indicating the targeting location of p73α/β siRNA. **B** The level of TAp73α, TAp73β, and actin proteins was measured in isogenic control, E13-KO cells transiently transfected with a scrambled or p73α/β siRNA. **C** Scratch assays were performed with isogenic control, E13-KO cells transiently transfected with a scrambled or p73α/β siRNA. The relative % of wound closure was shown below each image. **D** Isogenic control and E13-KO cells transiently transfected with a scrambled or p73α/β siRNA for 3 days, followed with mock or Erastin treatment for 8 h. Microscopic images were taken to show changes of cell morphology. **E** Isogenic control and E13-KO cells transiently transfected with a scrambled or p73α/β siRNA for 3 days, treated with or without Erastin (20 μM) for 8 h, followed by CellTiter-Glo to measure cell viability. The experiment was performed in triplicates and data were presented as mean ± SEM. *p < 0.05 by student *t*-test. **F** Cells were treated in (**E**), followed by RT-PCR analysis to measure the level of PTGS2, TFRC, LPCAT4, and actin mRNA.

To verify that p73β is necessary for growth suppression and sensitized ferroptosis observed in E13-KO cells (Figs. 1 and 4), an siRNA that targets E11 of *TP73*, called p73α/β siRNA, was designed and expected to only knock down p73α and p73β (Fig. 5A). As expected, the levels of p73α and p73β proteins were reduced by p73α/β siRNA in isogenic control and E13-KO H1299 cells (Fig. 5B). Next, scratch assay was performed using isogenic control and E13-KO H1299 cells transfected with a scrambled or p73α/β siRNA. We found that in isogenic control cells, knockdown of p73α led to increased cell migration (Fig. 5C), consistent with the data obtained from TAp73-KO H1299 cells (Fig. 1D, E). Notably, although E13-KO cells showed slower migration than isogenic control cells, knockdown of p73β in E13-KO cells led to enhanced cell migration (Fig. 5C), suggesting that p73β is required for the inhibition of cell migration in E13-KO cells. Next, we determined whether p73β is required for the enhanced ferroptosis as observed in E13-KO cells (Fig. 4). Indeed, we found that knockdown of p73α, the major isoform in isogenic control H1299 cells, enhanced cell viability under mock and Erastin-treated conditions (Fig. 5D, E). Importantly, knockdown of p73β in E13-KO cells, de-sensitized E13-KO cells to Erastin-induced ferroptosis (Fig. 5D, E). Consistent with this, the level of ferroptosis markers, including PTGS2, TFRC, and LPCAT4, were decreased by knockdown of p73α or p73β in both isogenic control and E13-KO cells, respectively (Fig. 5F). Since TAp73β is the major isoform expressed in E13-KO cells, thus, these data suggest that TAp73β is responsible for the inhibited cell migration and enhanced ferroptosis in E13-KO cells.

### CDO1 is a novel target of TAp73β and plays a role in TAp73β-dependent cell migration and ferroptosis

As shown above, we found that TAp73β is required for the enhanced ferroptosis observed in E13-KO cells (Fig. 5D, F). We also found that that E13-deficient mice were prone to chronic inflammation and liver steatosis (Fig. 4), likely due to enhanced ferroptosis. Thus, to understand how TAp73β regulates ferroptosis, RNA-seq analysis was performed to identify potential targets of TAp73β that are involved in ferroptosis by using two clones of isogenic control H1299 cells and two clones of E13-KO H1299 cells. Venn diagram showed that 260 genes were consistently altered in the two E13-KO clones (Supplementary Fig. 4A). Moreover, we cross-refenced the RNA-seq data with the Human Disease Ontology and found that E13-KO led to up-regulation of genes involved in several inflammatory diseases, such as pneumonia, nephritis, inflammatory bowel disease, and ulcerative colitis (Supplementary Fig. 4B). In addition, we found that the ferroptosis markers, such as PTGS2, TRFC, and LPCAT4, were up-regulated by E13-KO, which was also confirmed by qRT-PCR analysis (Supplementary Fig. 4C).

**Fig. 6:**
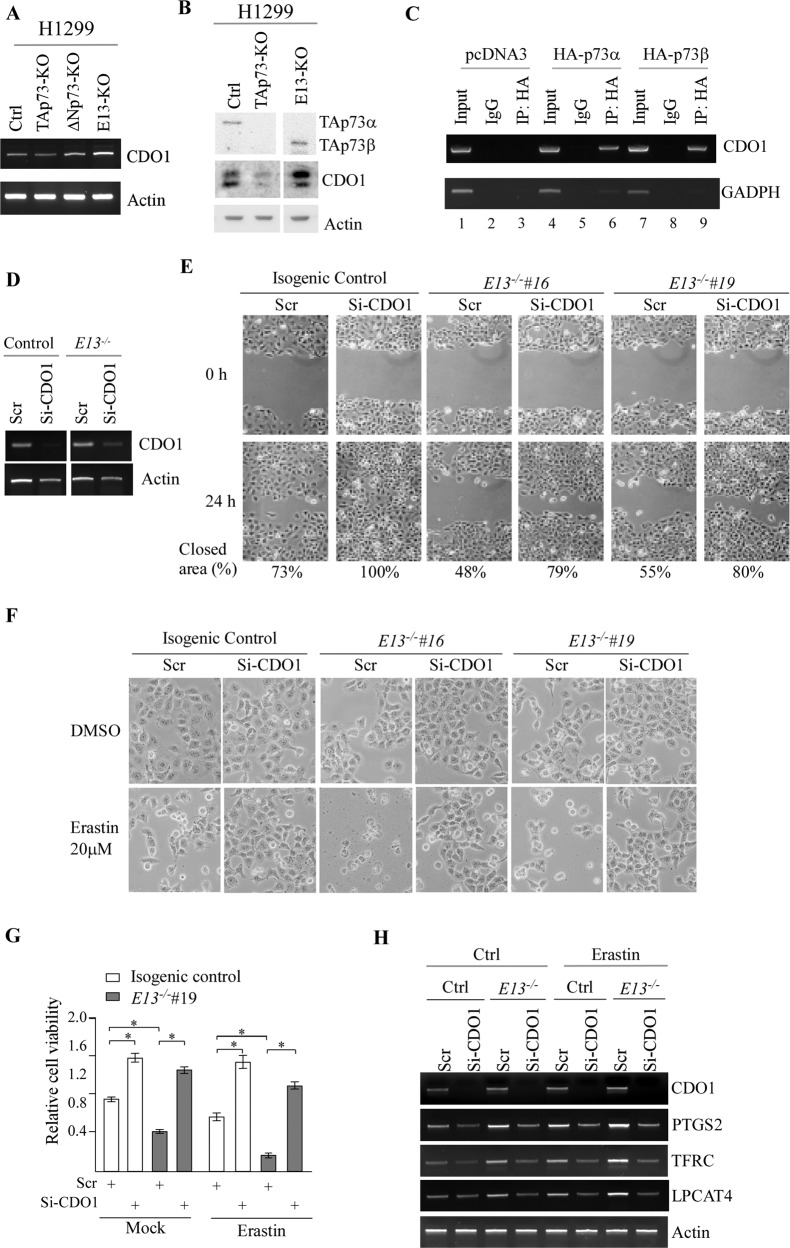
CDO1 is a novel target of TAp73β and plays a role in TAp73β-dependent cell migration and ferroptosis. **A** The level of CDO-1 and actin transcripts was measured in isogenic control, TAp73-KO and E13-KO H1299 cells. **B** The level of TAp73α, TAp73β, CDO-1 and actin was measured in isogenic control, TAp73-KO and E13-KO H1299 cells. **C** ChIP assay was performed with H1299 cells transiently transfected with an empty vector or a vector expressing HA-tagged TAp73α or TAp73β. **D** The level of CDO-1 and actin was measured in isogenic control and E13-KO H1299 cells transiently transfected with scrambled or CDO-1 siRNA. **E** Scratch assay was performed with isogenic control and E13-KO H1299 cells transiently transfected with scrambled or CDO-1 siRNA. The relative % of wound closure was shown below each image. **F** Isogenic control and E13-KO H1299 cells transiently transfected with scrambled or CDO-1 siRNA for 3 days, followed by mock or Erastin treatment for 8 h. Representative microscopic images were taken. **G** Isogenic control and E13-KO cells transiently transfected with a scrambled or CDO-1 siRNA for 3 days, treated with or without Erastin (20 μM) for 8 h, followed by CellTiter-Glo to measure cell viability. The experiment was performed in triplicates and data were presented as mean ± SEM. *p < 0.05 by student *t*-test. **H** Cells were treated in (**G**), followed by RT-PCR analysis to measure the level of CDO-1, PTGS2, TFRC, LPCAT4, and actin mRNAs.

Erastin-induced ferroptosis is known to induce cysteine depletion [46]. In addition, we showed above that E13-KO leads to decreased level of intracellular cysteine (Fig. 4D). Thus, to further understand the mechanism by which E13-KO, particularly TAp73β, induces ferroptosis, we focused on to identify a target of TAp73 involved in cysteine metabolism. Indeed, upon examining the RNA-seq data, we found that cysteine dioxygenase type 1 (CDO-1) was consistently up-regulated in two E13-KO H1299 cells when compared with isogenic control cells. CDO-1 is an enzyme that catalyzes the conversion of L-cysteine to cysteine sulfinic acid and thus, plays a critical role in cysteine catabolism by regulating the intracellular level of cysteine [47]. To confirm this, the level of CDO-1 transcripts was examined in isogenic control, TAp73-KO, ΔNp73-KO and E13-KO H1299 cells. We found that TAp73-KO led to a moderate decrease in CDO-1 transcripts (Fig. 6A). By contrast, CDO-1 transcript was increased mildly in ΔNp73-KO cells but markedly in E13-KO cells. Consistent with this, we found that CDO-1 protein was increased in E13-KO H1299 cells but decreased in TAp73-KO cells (Fig. 6B). Next, we searched the CDO-1 promoter for potential p53-response element and identified one located at -2032 to -2005 bp. To determine whether CDO-1 is transcriptionally regulated by p73, ChIP assay was performed with H1299 cells that were transiently transfected with a control vector or a vector expressing HA-tagged TAp73α or TAp73β. We found that the CDO-1 promoter was detected in both TAp73α and TAp73β immunoprecipitates (Fig. 6C, compare lane 3 with lane 6 and 9, respectively), suggesting that CDO-1 is a target of TAp73. As a negative control, TAp73α and TAp73β were unable to bind to the GAPDH promoter (Fig. 6C).

To determine whether CDO-1 plays a role in p73-mediated growth suppression and ferroptosis, CDO-1 siRNA was designed and then transiently transfected into isogenic control and E13-KO H1299 cells, followed by measuring cell migration and Erastin-induced ferroptosis. As expected, CDO-1 was efficiently knocked down by its siRNA (Fig. 6D). Importantly, we found that the ability of E13-KO cells to migrate was restored, reaching to the level observed in isogenic control cells without knockdown of CDO-1 (Fig. 6E). We also found that cell migration was increased by knockdown of CDO-1 in isogenic control cells. Moreover, we found that knockdown of CDO-1 made E13-KO cells resistance to Erastin-induced ferroptosis (Fig. 6F, G). Additionally, knockdown of CDO-1 made isogenic control cells resistant to Erastin-induced ferroptosis (Fig. 6F, G). In line with this, we found that knockdown of CDO-1 abrogated up-regulation of ferroptosis markers, including PTGS2, TFRC, and LPCAT4, in both isogenic control and E13-KO H1299 cells regardless of Erastin treatment (Fig. 6G). These findings were consistent with the observations above that knockdown of p73α/β promoted cell migration but de-sensitized cells to Erastin-induced ferroptosis (Fig. 5C–F). Together, these data indicate that CDO-1 is a mediator of TAp73β to regulate cell migration and ferroptosis by depleting intracellular cysteine.

## Discussion

Through alternative splicing, TP73 produces at least six C-terminal isoforms. Among them, TAp73β is found to be the most potent transactivator due to lack of the SAM and TID. However, the biological function of TAp73β remains largely uncharacterized due to lack of a physiologically relevant model system. In this study, by taking the advantage of CRISPR-Cas9 technology, we showed that deletion of *TP73* E13 led to predominant isoform switch from p73α to p73β in both cells and mice (Figs. 1 and 2). Thus, E13-deficient cells and mice are ideal models to study the biological function of p73β. We found that the rate of cell growth and migration in E13-KO cells is lower than that in isogenic control cells whereas the rate of cellular senescence in E13-KO MEFs is higher than that in WT MEFs (Figs. 1C, D and 2B). Moreover, we showed that knockdown of TAp73 but not ΔNp73 alleviated the inhibited cell migration in E13-KO cells (Fig. 1E). Since TAp73β is the predominant isoform in E13-KO cells whereas TAp73α is the predominant one in isogenic control cells and WT MEFs, we concluded that TAp73β is more potent than TAp73α in inducing growth suppression and senescence at a physiological relevant level. These data are in line with the previous in vitro studies that ectopic TAp73β is a potent inducer of growth suppression [3–5].

Recent studies have shown that p53 plays a critical role in ferroptosis in a context-dependent manner [48–50]. Thus, to better understand the biological functions of p73, we determined whether p73 plays a role in ferroptosis. We found that TAp73-KO cells were resistant, whereas ΔNp73-KO cells were sensitive, to Erastin-induced ferroptosis (Fig. 4). We also showed that E13-deficient cells, in which TAp73β is the main isoform expressed, were also sensitive to Erastin-induced ferroptosis. Notably, knockdown of p73α and/or p73β was able to attenuate Erastin-induced ferroptosis in both isogenic control and E13-KO cells (Fig. 5D–F). These data suggest that TAp73, particularly TAp73β, promotes, whereas ΔNp73 inhibits, ferroptosis. To understand how TAp73β induces ferroptosis, we identified CDO-1 as a novel target of p73 (Fig. 6). We also showed that knockdown of CDO-1 enhanced cell migration and inhibited ferroptosis in both isogenic control and E13-KO cells (Fig. 6D–G), which was consistent with a recent study indicating that CDO-1 can protect cells from ferroptosis [51]. So how does CDO-1 regulate ferroptosis? We postulate that through conversion of L-cysteine to cysteine sulfinic acid [52], CDO-1 controls the availability of intracellular cysteine and subsequently, the ferroptotic response [53]. In support of this notion, we found that the level of intracellular cysteine was much lower in E13-KO cells (high CDO-1) than that in isogenic control cells (low CDO-1) (Fig. 4D). Thus, further studies are worthwhile to elucidate whether ferroptosis mediated by the p73β-CDO-1 axis can be explored for cancer therapy.

To understand the biological function of p73β in vivo, we generated E13-KO mice in which p73β is the predominant isoform expressed. We would like to mention that while this manuscript was in preparation, an E13-deficient mouse model was independently generated by Melino’s group and showed the p73α is essential for proper hippocampal morphogenesis [34]. Additionally, they showed that p73β can fully substitute the function of p73α in regulating multiciliogenesis [54]. These data suggest that p73α and p73β have both common and distinct functions, which warrants further investigation. Thus, our study has been focused on the role of E13-deficiency in spontaneous tumors and chronic inflammation. Indeed, we found that *E13*-deficient mice were prone to spontaneous tumors, chronic inflammation, and liver steatosis when compared with WT mice in which p73α is the main isoform expressed (Fig. 3B–F and Supplementary Tables S1–3). In addition, when compared with *Trp73*-deficient mice, E13-deficient mice were more prone to chronic inflammation and liver steatosis (Fig. 3B–F and Supplementary Tables S2–3). These data strongly suggest that the enhanced ferroptosis by p73β may contribute to chronic inflammation and liver steatosis. Thus, further investigations are warranted to investigate whether CDO-1 is responsible for these pathological abnormalities in E13-deficient mice in vivo.

The tumor phenotypes in E13-deficient mice were puzzling as the tumor incidence was significantly higher in E13-deficient mice than that in WT mice, but no difference between E13-deficient and *Trp73*-deficient mice (Fig. 3B). These data indicate that although TAp73β is more potent than TAp73α in inducing growth suppression, TAp73β is insufficient to compensate for loss of p73α in tumor suppression. Several possibilities may explain this apparent paradox. First, it is possible that p73α, a strong transcriptional repressor, may repress a set of oncogenic targets to inhibit tumorigenesis. Second, owing to its strong transactivation potential, p73β may induce an array of genes, such as CDO-1, that sensitizes cells to ferroptosis and then elicits chronic inflammation, leading to tumorigenesis. Third, it remains possible that other p73 isoforms (i.e., δ and ε) increased by E13 skipping may have oncogenic activities. Therefore, further studies are needed to elucidate the common and distinct roles of various p73 C-terminal isoforms in tumor suppression.

In sum, our study provides new insights into the biological functions of p73β in vitro and in vivo. At cellular levels, p73β is a potent inhibitor of cell growth through the p73-CDO-1 axis. At organismal levels, p73β is a potent inducer of the inflammatory response and liver steatosis. Together, our data indicate that p73β is insufficient to compensate for loss of p73α in tumor suppression likely due to the adverse effects of the inflammatory response and liver steatosis on tumor suppression.

## Supplementary information

Reproducibility checklist

Uncut gel images

Supplemental Material Methods

Supplemental Figures

## Data Availability

The data that support the findings of this study are available in the methods and/or supplementary material of this article.
