# Supplementary material for: TP73 Isoform-specific disruption reveals a critical role of TAp73beta in growth suppression and inflammatory response
Source: Cell Death Dis. 2023 Jan 11;14(1):14. doi: 10.1038/s41419-022-05529-7 (PMC9834251; doi:10.1038/s41419-022-05529-7)

Figure 1B

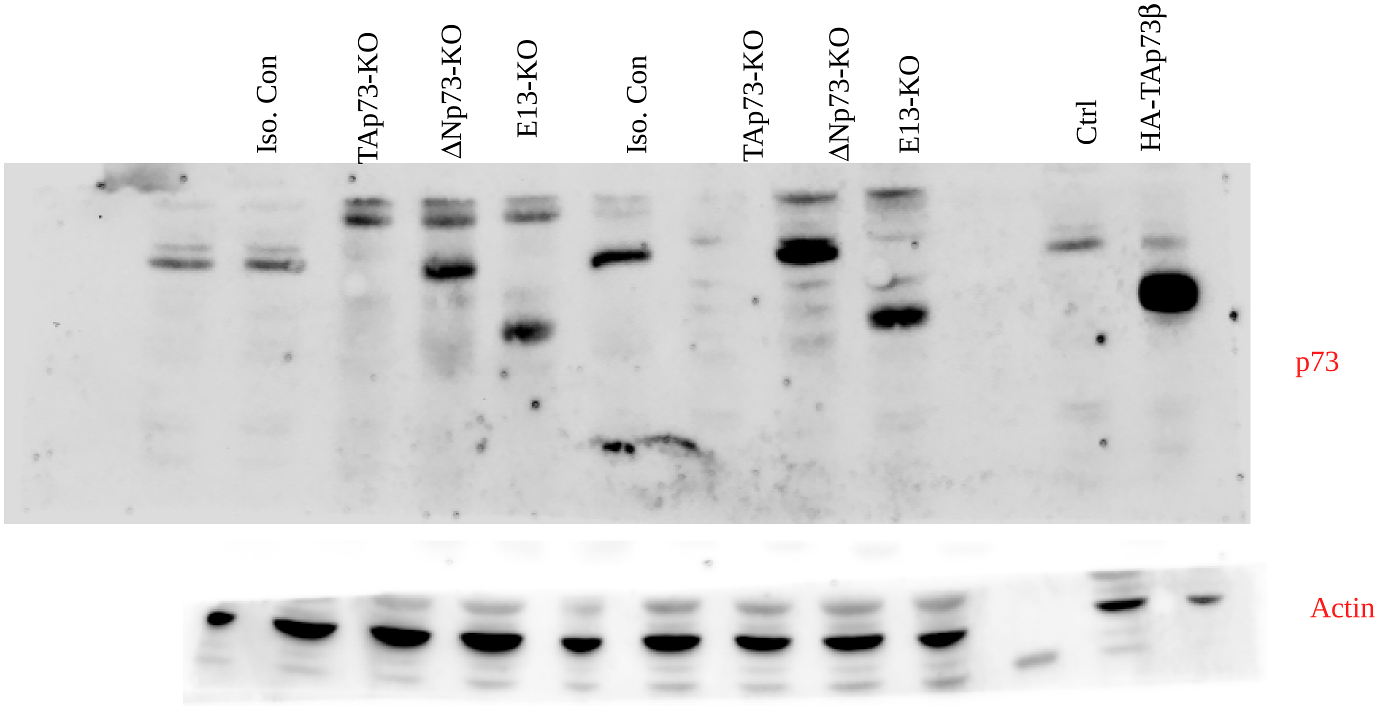
Iso. Con
TAp73-KO
ΔNp73-KO
E13-KO
Iso. Con
TAp73-KO
ΔNp73-KO
E13-KO
Ctrl
HA-TAp73β
p73
Actin

Figure 2C

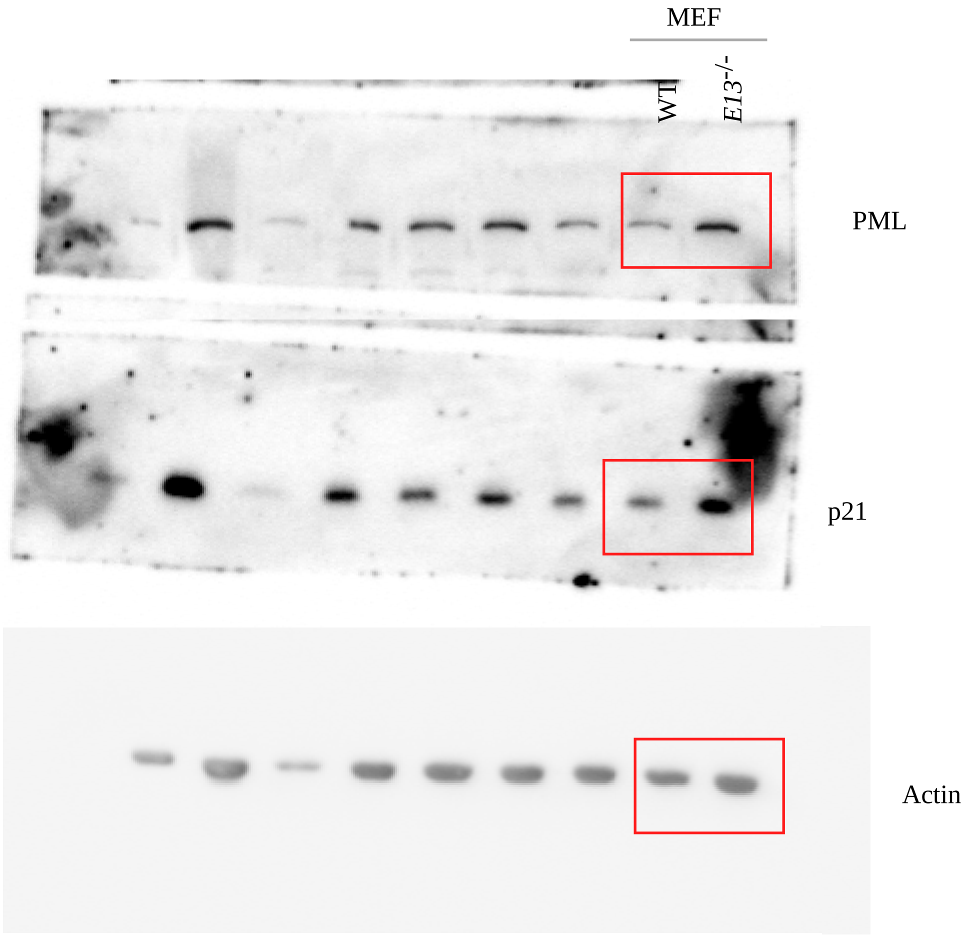

Figure 5B

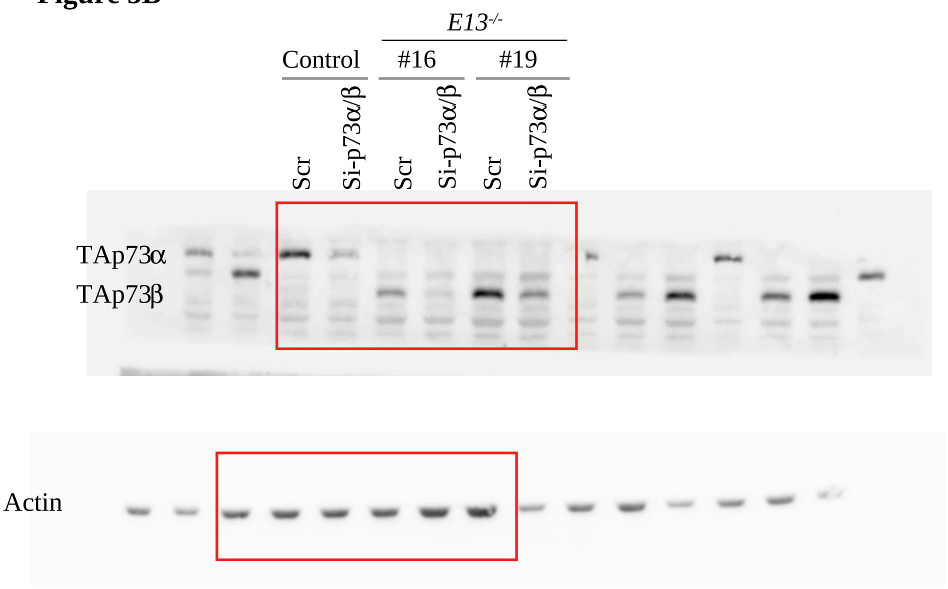
E13-/-
Control
#16
#19
Scr
Si-p73α/β
Scr
Si-p73α/β
Scr
Si-p73α/β
TAp73α
TAp73β
Actin

Figure 6A

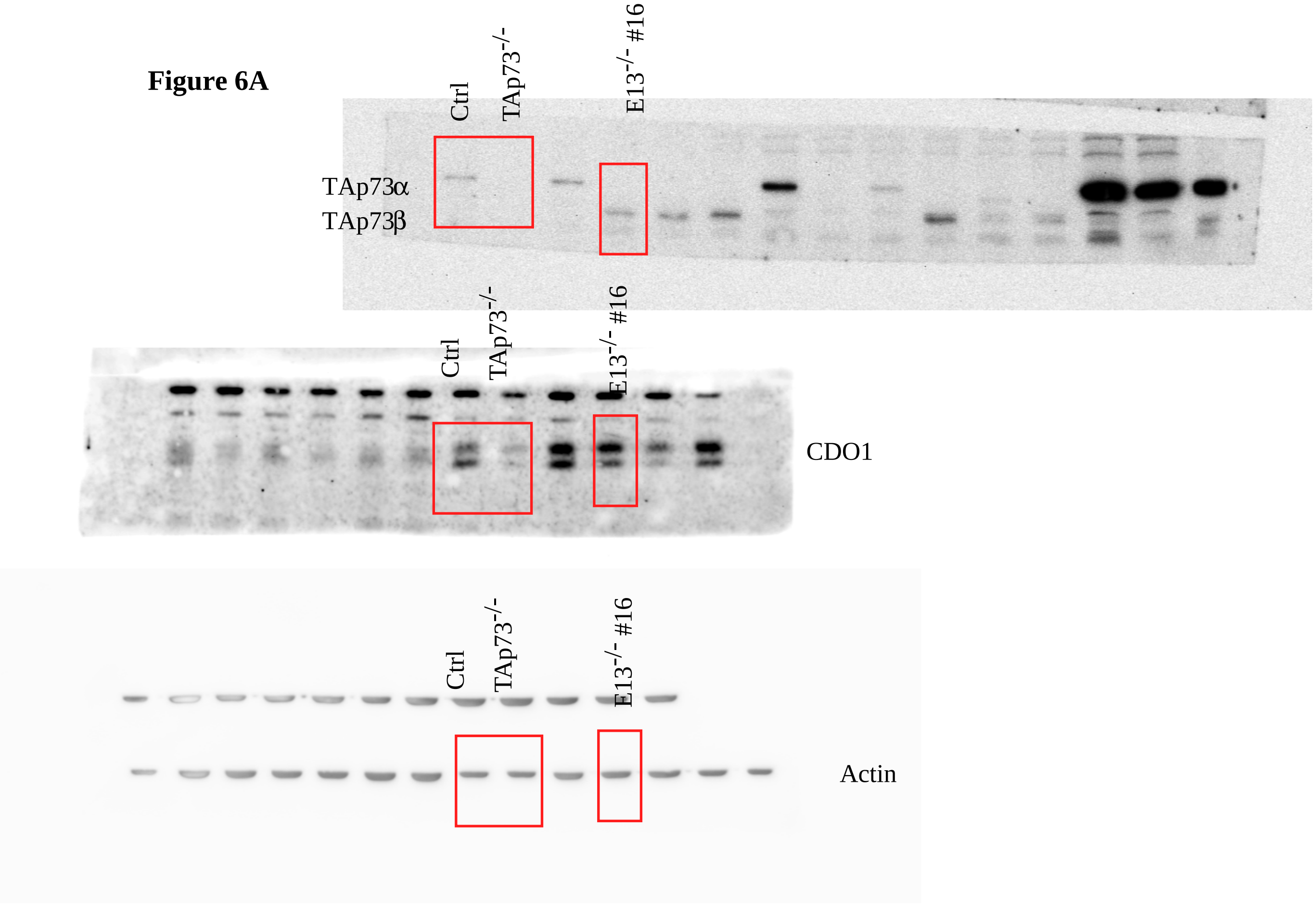

Ctrl
TAp73$^{-/-}$
E13$^{-/-}$ #16
TAp73α
TAp73β
Ctrl
TAp73$^{-/-}$
E13$^{-/-}$ #16
CDO1
Ctrl
TAp73$^{-/-}$
E13$^{-/-}$ #16
Actin

Supplemental Figure 1E

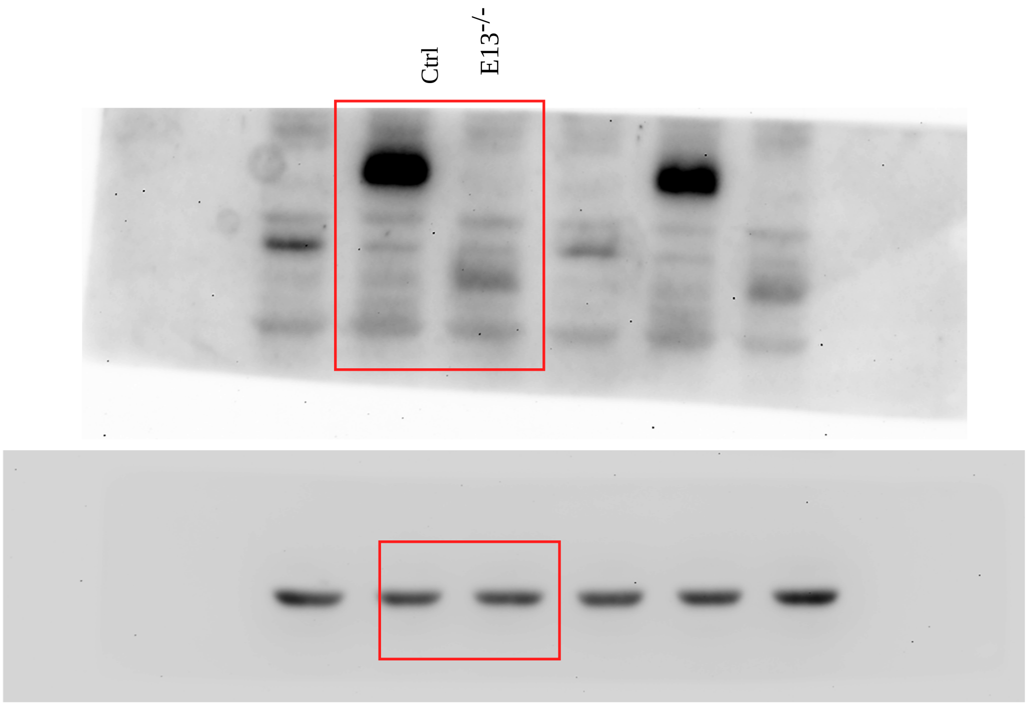

Supplemental Figure 1F

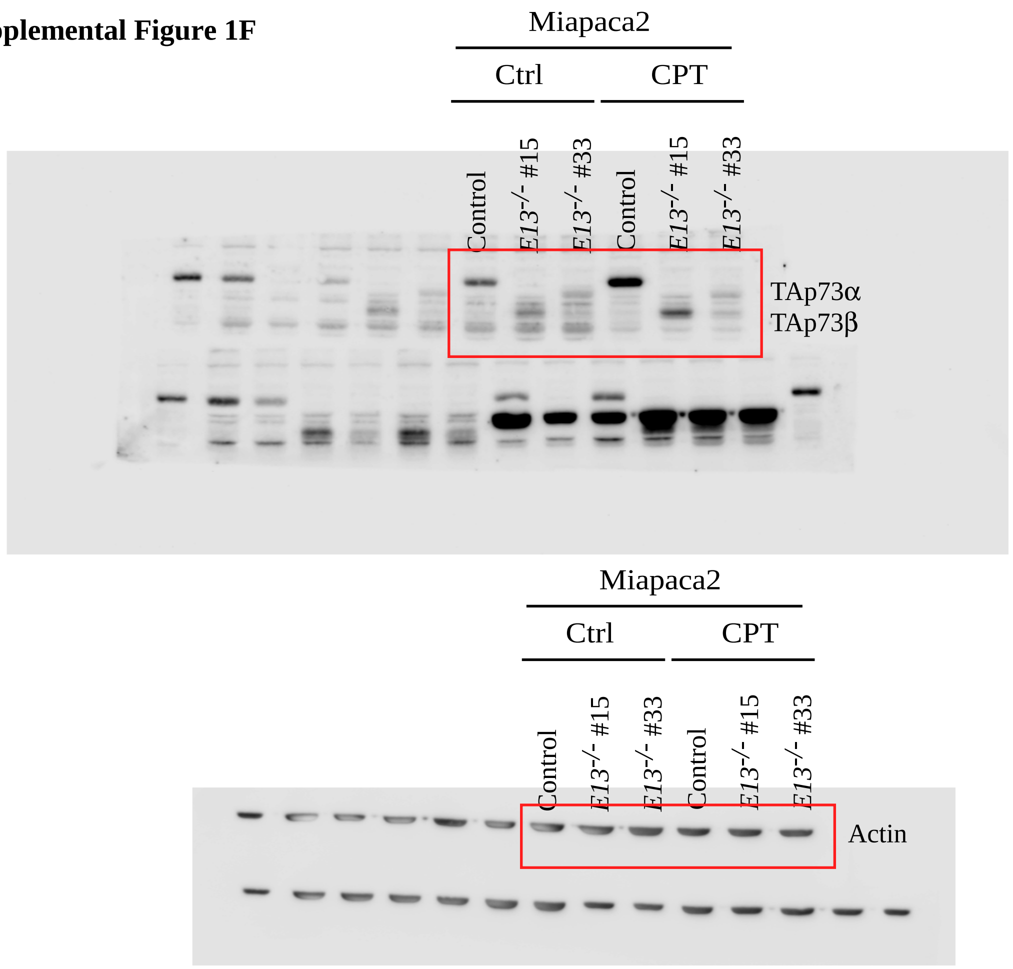

Supplemental Figure 1H

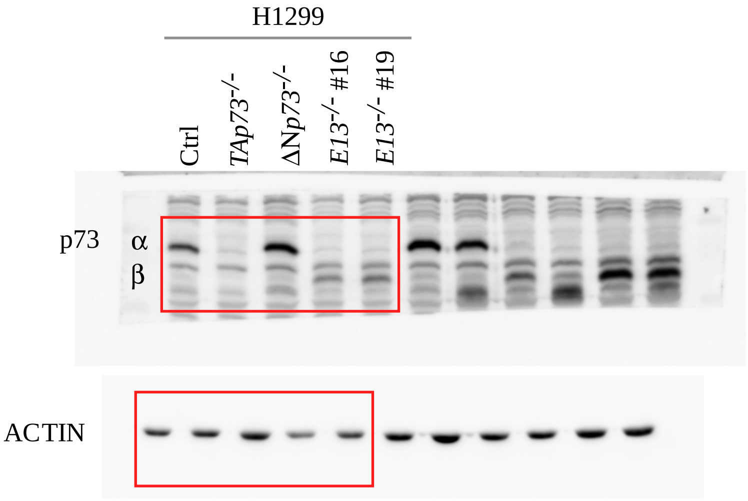

Supplemental Figure 1I

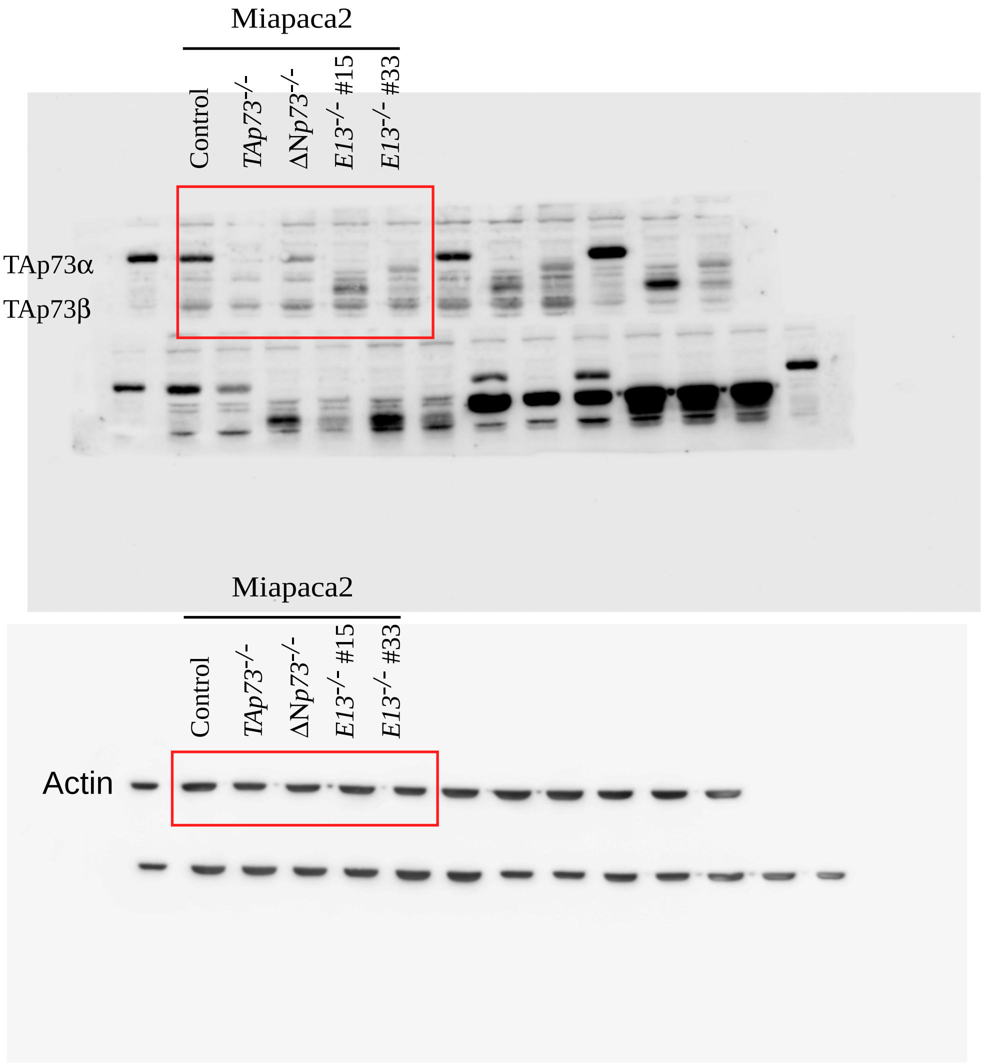

Supplement: Supplementary file 2 — Uncut gel images [file 41419_2022_5529_MOESM2_ESM.pdf]
