# Supplementary material for: TP73 Isoform-specific disruption reveals a critical role of TAp73beta in growth suppression and inflammatory response
Source: Cell Death Dis. 2023 Jan 11;14(1):14. doi: 10.1038/s41419-022-05529-7 (PMC9834251; doi:10.1038/s41419-022-05529-7)
Supplement: Supplementary file 3 — Supplemental Material Methods [file 41419_2022_5529_MOESM3_ESM.pdf]

Supplemental Material Methods

Untargeted metabolomics by HILIC-MS for biogenic amines

Sample Preparation Metabolites were extracted from 1x10^6 cells using 1 mL of degassed, −20 ◦C cold mixture of ACN:IPA:H2O (3:3:2, v/v/v). Samples were vortexed for 10 seconds, shaken for 5 min and then centrifuged for 2 min at 14,000 rcf. Two 450 µL supernatant aliquots were transferred to new tubes. One tube was stored as a backup aliquot and another was dried in a SpeedVac concentrator. Sample were re-suspended with 100 µL of ACN:H2O (80:20, v/v) which contained deuterium labeled internal standards prior to injection.

Data Acquisition 5 µL sample aliquots were injected on a Waters Acquity UPLC BEH Amide column (150 mm length × 2.1 mm i.d.; 1.7 µm particle size) maintained at 45 ◦C. A Waters Acquity VanGuard BEH Amide pre-column (5 mm × 2.1 mm i.d.; 1.7 µm particle size) was used as guard column. Mobile phase A was 100% LC-MS grade H2O with 10 mM ammonium formate and 0.125% formic acid and mobile phase B was 95:5 v/v ACN:H2O with 10 mM ammonium formate and 0.125% formic acid. Gradient was started at 100% (B) for 2 min, 70% (B) at 7.7 min, 40% (B) at 9.5 min, 30% (B) at 10.25 min, 100% (B) 14 at 12.75 min, and isocratic until 16.75 min. The column flow was 0.4 mL/min.

Agilent 1290 Infinity UHPLC coupled with SCIEX TripleTOF® 6600 QTOF was used for LC-MS/MS analysis. The parameters were: MS1 mass resolution, 35000; curtain gas, 35; ion source gas 1, 50 psi; ion source gas 2, 50 psi; temperature, 350 °C; ion spray voltage, 4.5 kV(ESI+) and 3.5 kV (ESI-); declustering potential, 80 V; MS1 accumulation time, 100 ms; MS1 mass range, m/z 60–900; top-10 DDA; MS/MS accumulation time, 10 ms; collision energy, 25 eV; collision energy spread, 15 eV.

Data Processing Raw data files were processed with MS-DIAL v4.60. For each m/z value ion chromatogram was extracted with m/z thresholds of 0.01 Da and retention time threshold of 0.10 min. Apex of the extracted ion chromatograph was used as peak height value. Metabolite annotation was conducted by matching accurate mass (<10 mDa), retention time (0,1min), and MS/MS. MS/MS references are from NIST17 MS/MS library and MoNA database (massbank.us), in addition to a specific in-house HILIC-retention time MS/MS mass spectral library of 1200 authentic standards. Data were normalized using a random forest algorithm-based signal correction method.

RNA-seq analyses

RNA-seq libraries from 1 µg of total RNA were prepared by using Illumina Tru-Seq RNA Sample Prep Kit, according to the manufacturer's instructions. Sequencing was performed on an an Illumina NovaSeq 6000 platform. Paired-end clean reads in FastQ format generated by the Illumina pipeline were aligned to the reference human genome (UCSC hg19) by Burrows-Wheeler Aligner (BWA) to get the original mapping results stored in BAM format (1). The stringtie '-eB' and '-A' were used to estimated raw read counts and gene expression abundance using the method of Fragments Per Kilobase of exon model per Million mapped fragments

(FPKM), respectively. DESeq2 was used for transcript assembly, quantification of normalized gene and isoform expression, and analysis of different expression. Gene Set Enrichment Analysis (GSEA v.3.0) was applied to compare genes based on the log2 fold changes.

References

1. Li H, Durbin R. Fast and accurate short read alignment with Burrows-Wheeler transform. Bioinformatics. 2009;25(14):1754-60.
