# Supplementary material for: TP73 Isoform-specific disruption reveals a critical role of TAp73beta in growth suppression and inflammatory response
Source: Cell Death Dis. 2023 Jan 11;14(1):14. doi: 10.1038/s41419-022-05529-7 (PMC9834251; doi:10.1038/s41419-022-05529-7)
Supplement: Supplementary file 4 — Supplemental Figures [file 41419_2022_5529_MOESM4_ESM.pdf]

Supplemental Figure 1

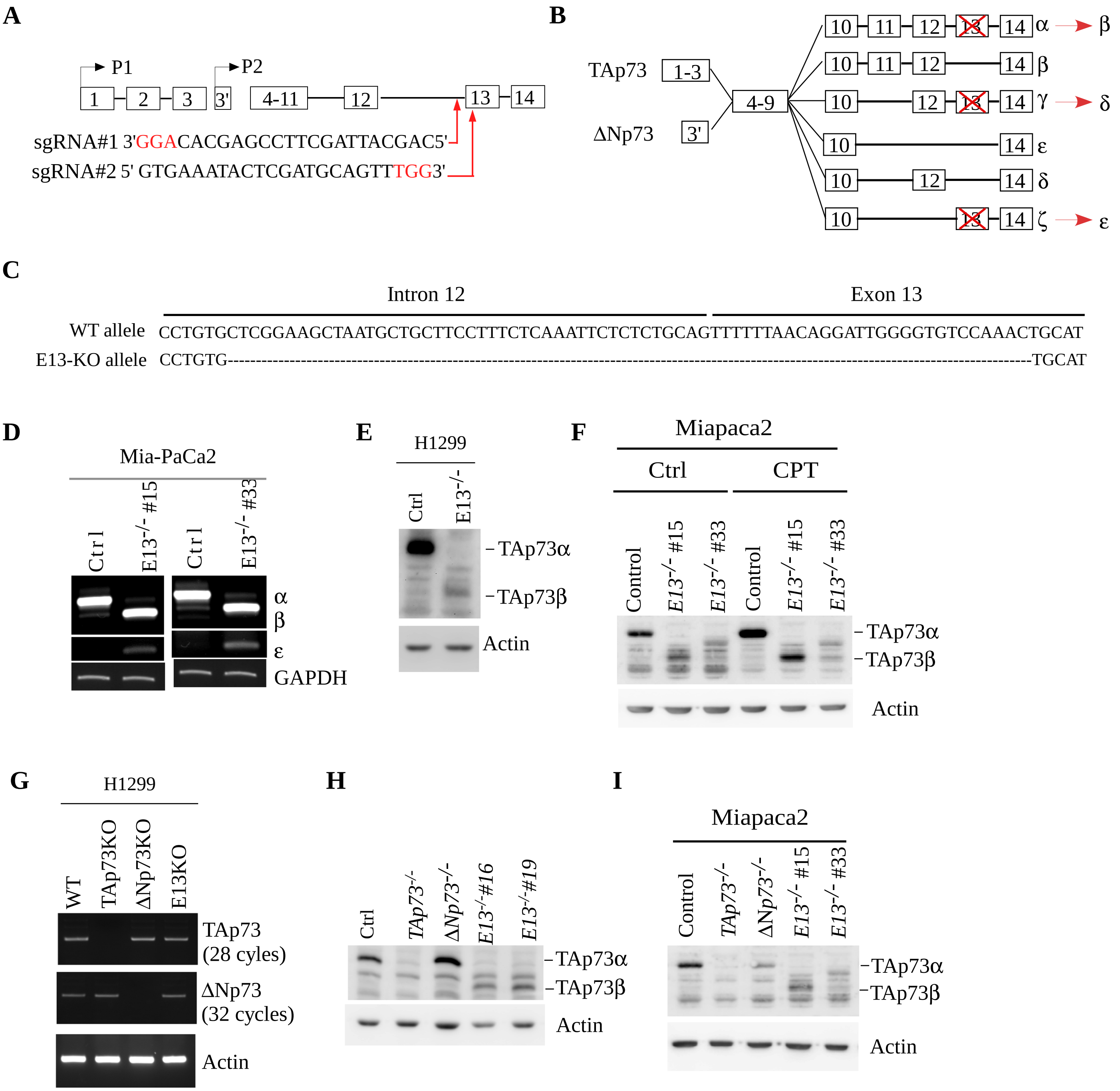

Supplemental Figure 1 Loss of E13 leads to isoform switch from p73α to p73β.

(A) Schematic representation of the location and sequence for two guide RNAs that target TP73 E13.
(**B**) Schematic representation of p73 isoform switch resulting from E13 deletion.
(**C**) The level of p73α, p73β, p73ε, and GAPDH transcripts was measured in isogenic control and E13-KO Mia-PaCa2 cells.
(**D**) The level of TAp73α, TAp73β, and actin proteins was measured in isogenic control and E13-KO Mia-PaCa2 cells mock-treated or treated with camptothecin.
(**E**) The level of TAp73α, TAp73β, and actin proteins was measured in isogenic control, TAp73-KO, ΔNp73-KO and E13-KO Mia-PaCa2 cells.
(**F**) Isogenic control, *TAp73*$^{-/-}$, Δ*Np73*$^{-/-}$, and *E13*$^{-/-}$ H1299 cells were mock-treated or treated with Erastin for 10 hours, followed by CellTiter-Glo assay to measure the cell viability.
(**G**) The level of PTGS2, TFRC, LPCAT4 and actin mRNA was measured in isogenic control, *TAp73*$^{-/-}$, Δ*Np73*$^{-/-}$, and *E13*$^{-/-}$ H1299 cells mock-treated or treated with Erastin.
(H) The level of intracellular cysteine was measured in isogenic control and E13-KO H1299 cells using SCIEX TripleTOF system

Supplemental Figure 2

A

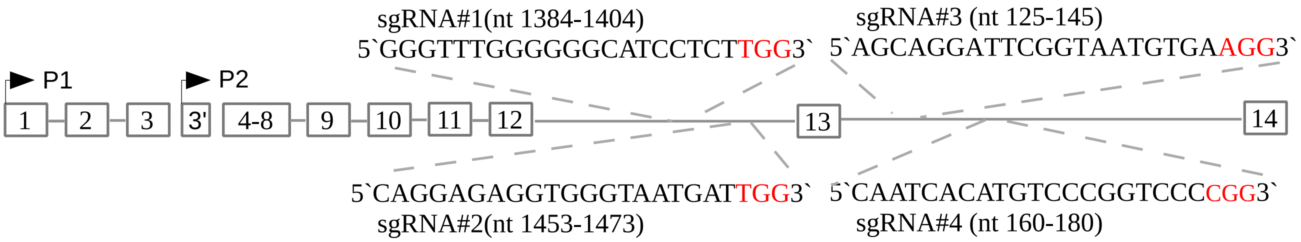

B

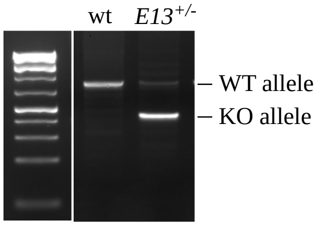

Supplemental Figure 2 Strategy to generate E13-deficient mice.
(**A**) Schematic representation of the strategy to generate E13-deficient mouse model and the location of four guide RNAs.
(B) Genotyping to verify WT and *E13*$^{+/-}$ mice.

Supplemental Figure 3

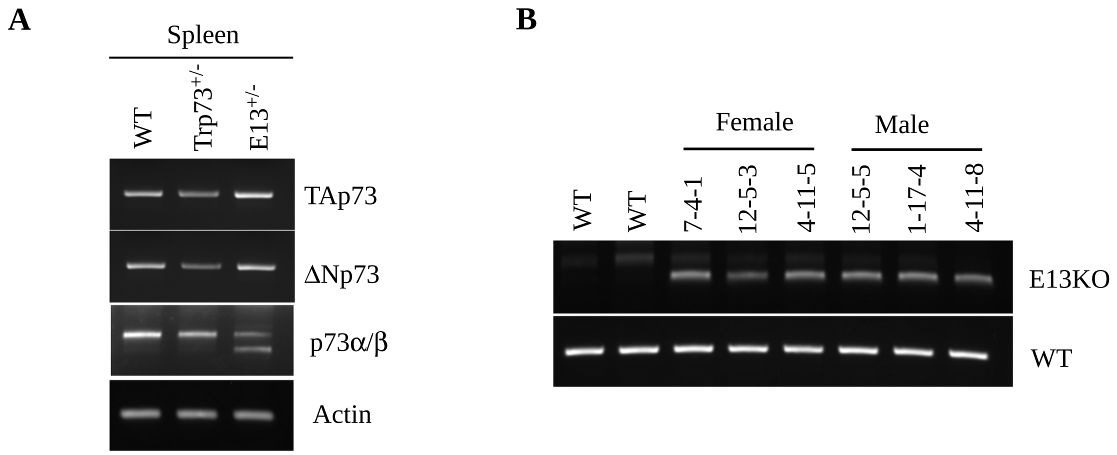

Supplemental Figure 3

(A) The level of TAp73, ΔNp73, p73α/β and actin transcript was measured in the spleen tissues from wildt type (WT), *Trp73*$^{+/-}$, and *E13*$^{+/-}$ mice.

(**B**) Gentoyping results indicated that there was no loss of heterozygosity in *E13*$^{+/-}$ mice.

Supplemental Figure 4

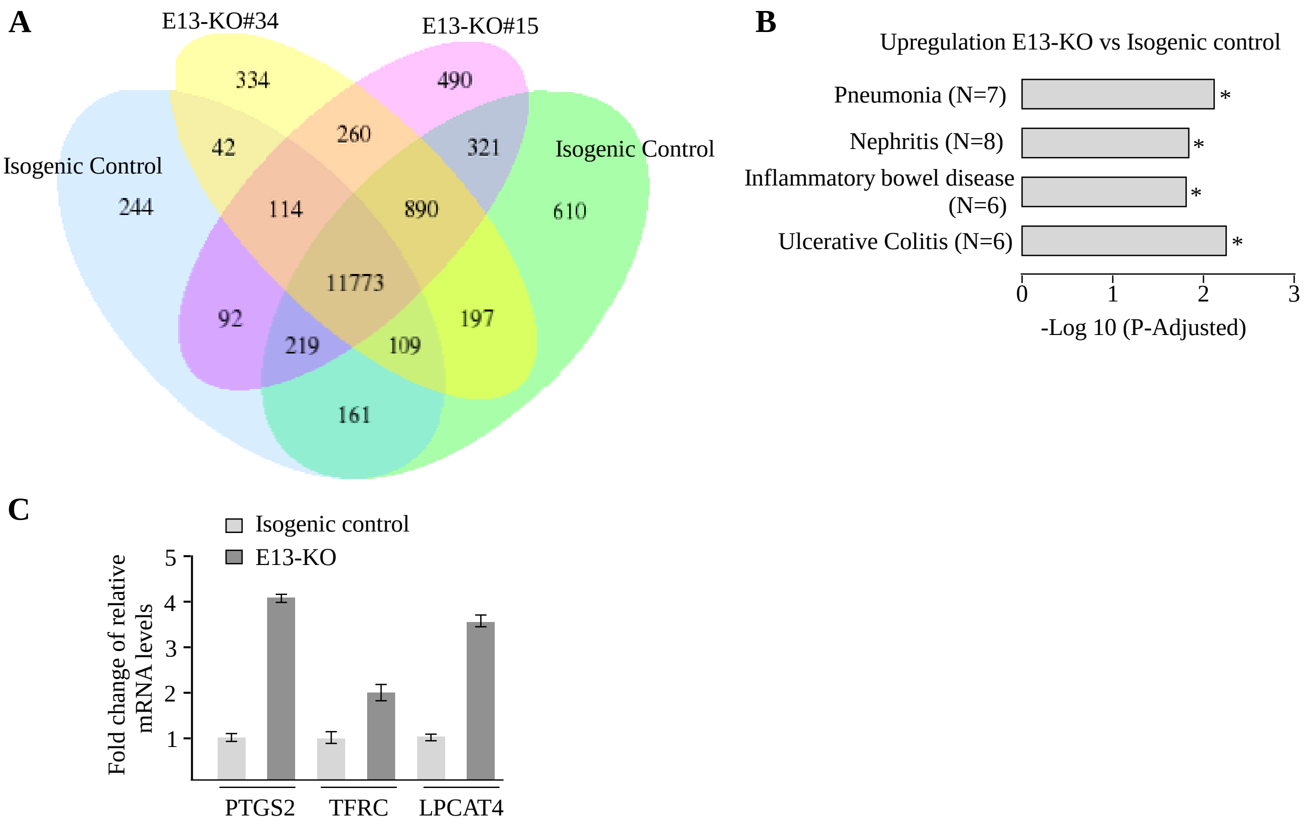

Supplemental Fig. 4

(**A**) Venn diagram indicated the numbers genes of that are significant differentially expressed or overlapped among two isogenic control and two E13-KO H1299 cells.
(**B**) Disease ontology enrichment analysis indicates that several pathways related to inflammatory response were up-regulated by loss of E13.
(**C**) RNA-seq analysis indicated relative fold change of PTGS2, TFRC, and LPCAT4 mRNA levels in isogenic control and E13-KO H1299 cells

Supplemental Table S1: Wild type (WT) mice (n=56) - survival time, tumor spectrum, steatosis, inflammation, and other abnormalities

ID	Gender	Survival (Wks)	Tumor	Steatosis	inflammation	Other abnormalities
5	F	134	-	-	-	-
7	F	117	-	-	-	-
16	F	100	-	-	-	-
22	F	109	-	-	-	-
25	F	109	-	-	-	-
44	F	90	-	-	-	-
55	F	104	T-LBL	-	-	-
64	F	120	-	-	-	-
2	M	127	-	-	-	-
3	M	117	-	-	Liver	-
12	M	127	-	-	-	-
13	M	127	-	-	-	-
20	M	122	-	-	-	-
23	M	122	-	-	-	-
26	M	127	-	-	Liver/Salivary gland	-
34	M	124	DLBCL	-	-	-
37	M	134	-	-	-	-
62	M	128	-	-	-	-
45	M	133	-	-	-	-
49	M	117	-	-	-	-
50	M	113	T-LBL/ DLBCL	-	-	-
56	M	117	DLBCL	-	-	-
59	M	119	DLBCL	-	-	-
65	M	106	-	-	-	-
69	M	102	DLBCL	-	-	SH
70	M	103	-	-	-	TH
71	M	90	-	-	-	-
1-24-2	M	83	-	Y	-	-
2-15-2	F	140	Lymphoma	-	-	EMH in liver
2-19-6	M	132	-	-	Pancreas	EMH in Spleen
2-19-2	F	143	-	-	-	EMH in Spleen
3-11-3	M	129	-	-	-	-
3-28-5	F	85	-	Y	-	EMH in spleen
3-9-7	M	129	-	-	-	-
5-12-3	F	99	Lymphoma	-	-	EMH in Spleen
7-9-9	F	116	-	-	-	-
8-2-6	M	120	-	-	-	-
10-24-7	F	130	-	-	Pancreas	EMH in spleen
10-26-6	F	129	Histiocytic sarcoma	-	-	EMH in spleen/liver
11-10-7	M	121	-	-	-	-
11-7-3	F	121	-	-	-	-
11-29-2	F	144	-	Y	-	EMH in spleen
12-2-4	F	113	-	-	-	-
12-20-7	F	115	-	-	-	EMH in Spleen
11-9-6	F	96	DLBCL	-	Skin	-
1-19-1	F	86	-	-	Skin	-
11-10-15	F	105	-	-	Skin/Pancreas	-
12-25-6	M	111	-	-	-	Hepatocirrhosis
1-19-5	F	109	Lymphoma	-	-	-
7-22-4	M	86	-	-	-	-
7-22-7	M	126	-	-	Kidney	-
11	M	111	N/A			Found dead
42	M	111	N/A			Found dead
43	M	107	N/A			Found dead
46	M	117	N/A			Found dead
52	M	101	N/A			Found dead

These mice were from published studies (Yang et al, 2017, PNAS, 114 (43) 11500-11505; Zhang et al, 2017, Genes & Dev, 31:1243-56)
DLBCL:Diffuse large B-cell lymphoma; EMH: Extramedullary hematopoiesis; TH: Thymic hyperplasia; SH: Splenic hyperplasia
N/A: not applicable

Supplemental Table S2: *Trp73*$^{+/-}$ mice (n=30)- survival time, tumor spectrum, steatosis, inflammation, and other abnormalities

ID#	Gender	Survival (Wks)	Tumor	Steatosis	Inflammation	Other abnormalities
1-30-11	F	82	-	-	Liver/Salivary gland/ kidney/ pancreas	SH/ EMH
6-7-14	M	43	-	-	Liver/lung/Kidney/ Salivary gland/skin	SH / EMH / TH
9-23-1	F	77	Hepatoma	-	Liver/pancreas/kidney	SH
12-14-3	F	46	-	-	Skin/lung/kidney/liver/salivary gland	SH / EMH
5-27-14	M	65	-	-	Liver/salivary gland	SH / EMH
9-13-11	F	69	-	-	Liver/Salivary gland/lung/kidney	SH / EMH
11-6-1	F	55	T-LBL	-	Liver/Kidney/Salivary gland/pancreas/lung	SH / EMH
5-3-5	F	105	-	Y	kidney/liver/pancreas/salivary gland	SH / EMH / TH
11-6-5	F	88	DLBCL/Hemangioma	Y	Liver/Salivary gland	SH / EMH / TH
5-13-2	M	117	-	Y	Liver/Salivary gland/Kidney	SH / EMH / TH
11-13-1/5	M	99	-	-	Liver, Salivary gland	-
10-13-12	F	89	Lymphoma	-	-	-
11-5-7	F	102	DLBCL/Lymphoma	-	Kidney/Liver/Cecum	SH
6-11-6	M	108	Lymphoma	-	Liver	SH
10-3-11	F	99	-	-	Liver/salivary gland/lung	SH / TH
10-22-4	F	86	Histiocytic sarcoma	-	Kidney/Salivary gland	-
5-28-16	F	120	Lymphoma	-	-	-
11-13-4	F	74	Lymphoma	-	-	-
12-14-1	F	103	-	-	Liver/pancreas/Salivary gland	SH
1-25-4	F	94	DLBCL	-	-	-
1-15-8	F	69	-	-	Liver/Kidney/salivary gland	SH
10-31-4	F	88	Lymphoma	-	Liver/Kidney/Lung/Salivary gland	SH/EMH
11-6-4	F	62	Gastric Adenoma	-	Lung/Salivary gland/Liver/Kidney	SH/ EMH
9-16-5	F	76	-	-	Kidney/Liver/Salivary gland	SH/EMH<br>Liver multifocal necrosis
6-22-4	F	80	-	-	Kidney/Lung/liver	SH/EMH<br>Liver multifocal necrosis
4-19-1	F	102	-	-	Liver/Heart	SH/FH
10-31-5	F	99	DLBCL	Y	Kidney/Lung/Salivary gland	-
10-9-2	M	101	N/A			Found dead
2-10-3	M	57	N/A			Found dead
3-10-3	M	108	N/A			Found dead

DLBCL:Diffuse large B-cell lymphoma; EMH: Extramedullary hematopoiesis; SH: Splenic hyperplasia; TH: Thymic hyperplasia; FH: Follicular hyperplasia

The data of the first 25 mice were from published studies (PNAS, 2019;116(48):24259-24267; J Pathol 2020; 251:284–296)

Supplemental Table S3: *E13*$^{+/-}$ mice (n=31) - survival time, tumor spectrum, inflammation, and other abnormalities

ID	Gender	Survival (Wks)	Tumor	inflammation	Liver steatosis	Other abnormalities
1-17-4	M	87	-	Kidney/Liver/Lung/Pancreas/Salivary gland	Y	SWPH/EMH in spleen
12-24-5	M	90	-	Kidney/Lung/Liver/Pancreas/Salivary gland/muscle	Y	SWPH/EMH in spleen
12-5-5	M	93	-	Kidney/Liver/Lung/Pancreas/Salivary gland	Y	SWPH/EMH in spleen
1-17-2	M	87	-	Kidney/Lung/Pancreas/Salivary gland	-	SWPH/TH
3-1-3	F	87	DLBCL	Kidney/Liver/Lung/Pancreas/Salivary gland	Y	TH/FH
5-17-11	F	77	-	Kidney/Liver/Lung/Pancreas/Salivary gland	Y	SWPH/EMH in spleen/FH
4-11-8	M	75	-	Kidney/Lung/Salivary gland	-	SWPH/EMH in spleen
5-17-5	M	62	-	Kidney/Lung/Salivary gland	-	SWPH/EMH in spleen
7-4-1	F	95	Lymphoma	Kidney/Liver/Lung/Salivary gland	-	SWPH/EMH in spleen/FH/ fibrohistiocytic granuloma
4-11-9	F	109	DLBCL	Kidney/Lung/Pancreas/Liver	-	EMH in spleen
12-26-3	M	64	DLBCL	Kidney/Lung/Pancreas/Liver	-	SWPH/EMH in spleen
12-26-2	M	64	DLBCL/Adenoma	Kidney/Liver/Lung/Pancreas/Salivary gland/GI	-	EMH in spleen
12-5-3	F	114	-	Kidney/Liver	Y	SWPH/EMH in spleen
11-12-2	F	117	DLBCL	Kidney/Liver/Salivary gland	-	EMH in spleen
5-17-8	M	134	Lymphoma/ Papillary adenocarcinoma	Lung/Liver/Salivary gland	-	EMH in spleen and Liver
4-11-5	F	119	DLBCL	Kidney/Liver/Salivary gland	Y	EMH in spleen
6-7-4	F	84	-	Kidney/Liver/Lung/Salivary gland	Y	SWPH/EMH in spleen/FH
8-17-8	F	121	-	Kideny/Liver/Lung	Y	SWPH/EMH in spleen/TH/FH
5-17-9	F	116	SCS/Lymphoma	Kidney/Lung/Liver/Salivary gland	Y	EMH in spleen
12-5-4	M	89	-	-	-	SWPH/EMH in spleen
8-17-9	F	118	DLBCL	Kidney/Liver/Lung/Salivary gland	-	EMH in spleen and Liver
1-23-8	F	107	DLBCL	Brown fat/Salivary gland	Y	EMH in spleen
8-17-3	F	121	-	Kidney/Lung/Liver/Pancreas/Salivary gland	-	SWPH/EMH in spleen
1-23-9	F	107	DLBCL	Kideny/Liver/Pancreas	-	-
6-12-2	F	130	-	Kidney/Lung/Liver/Salivary gland	-	SWPH/EMH in spleen
7-4-3	F	133	-	Kidney/Lung/Liver/Pancreas/Salivary gland	-	SWPH/EMH in spleen
7-18-3	M	102	-	Kidney/Liver/Salivary gland	Y	EMH in spleen/SWPH
6-7-6	M	107	Lymphoma	Salivary gland	-	EMH in spleen/SWPH
7-18-5	M	102	-	Lung/Liver/Salivary gland	Y	EMH in spleen/SWPH
8-17-4	F	89	N/A			Found dead
12-5-2	F	91	N/A			Found dead

DLBCL:Diffuse large B-cell lymphoma; EMH: Extramedullary hematopoiesis; SWPH: Spleen white pulp hyperplasia; SCS: Spindle cell sarcoma; TH: Thyrmic hyperplasia; FH: Follcular hyperplasia
